# Single-cell RNA-seq Reveals the Inhibitory Effect of Methamphetamine on Liver Immunity with the Involvement of Dopamine Receptor D1

**DOI:** 10.1093/gpbjnl/qzae060

**Published:** 2024-08-28

**Authors:** Jin-Ting Zhou, Yungang Xu, Xiao-Huan Liu, Cheng Cheng, Jing-Na Fan, Xiaoming Li, Jun Yu, Shengbin Li

**Affiliations:** Key Laboratory of National Health Commission for Forensic Sciences, Xi’an Jiaotong University, Xi'an 710061, China; National Biosafety Evidence Foundation, Bio-evidence Sciences Academy, Xi'an Jiaotong University, Xi'an 710115, China; Department of Cell Biology and Genetics, School of Basic Medical Sciences, Xi’an Jiaotong University Health Science Center, Xi’an 710061, China; Department of Respiratory and Critical Care Medicine, The Second Affiliated Hospital of Xi'an Jiaotong University, Xi'an 710004, China; Key Laboratory of National Health Commission for Forensic Sciences, Xi’an Jiaotong University, Xi'an 710061, China; National Biosafety Evidence Foundation, Bio-evidence Sciences Academy, Xi'an Jiaotong University, Xi'an 710115, China; Key Laboratory of National Health Commission for Forensic Sciences, Xi’an Jiaotong University, Xi'an 710061, China; National Biosafety Evidence Foundation, Bio-evidence Sciences Academy, Xi'an Jiaotong University, Xi'an 710115, China; National & Local Joint Engineering Research Center of Biodiagnosis and Biotherapy, Precision Medical Institute, The Second Affiliated Hospital of Xi'an Jiaotong University, Xi'an 710004, China; National Biosafety Evidence Foundation, Bio-evidence Sciences Academy, Xi'an Jiaotong University, Xi'an 710115, China; OneHealth Technology Company, Xi'an 710000, China; Key Laboratory of National Health Commission for Forensic Sciences, Xi’an Jiaotong University, Xi'an 710061, China; National Biosafety Evidence Foundation, Bio-evidence Sciences Academy, Xi'an Jiaotong University, Xi'an 710115, China

**Keywords:** Methamphetamine chronic exposure, Liver, Immunity, Dopamine receptor D1, Single-cell RNA sequencing

## Abstract

Methamphetamine (METH) is a highly addictive psychostimulant that causes physical and psychological damage and immune system disorder, especially in the liver which contains a significant number of immune cells. Dopamine, a key neurotransmitter in METH addiction and immune regulation, plays a crucial role in this process. Here, we developed a chronic METH administration model and conducted single-cell RNA sequencing (scRNA-seq) to investigate the effect of METH on liver immune cells and the involvement of dopamine receptor D1 (DRD1). Our findings reveal that chronic exposure to METH induces immune cell identity shifts from IFITM3^+^ macrophage (Mac) and CCL5^+^ Mac to CD14^+^ Mac, as well as from FYN^+^CD4^+^ T effector (Teff), CD8^+^ T, and natural killer T (NKT) to FOS^+^CD4^+^ T and RORα^+^ group 2 innate lymphoid cell (ILC2), along with the suppression of multiple functional immune pathways. DRD1 is implicated in regulating certain pathways and identity shifts among the hepatic immune cells. Our results provide valuable insights into the development of targeted therapies to mitigate METH-induced immune impairment.

## Graphical Abstract

**Graphical Abstract:**
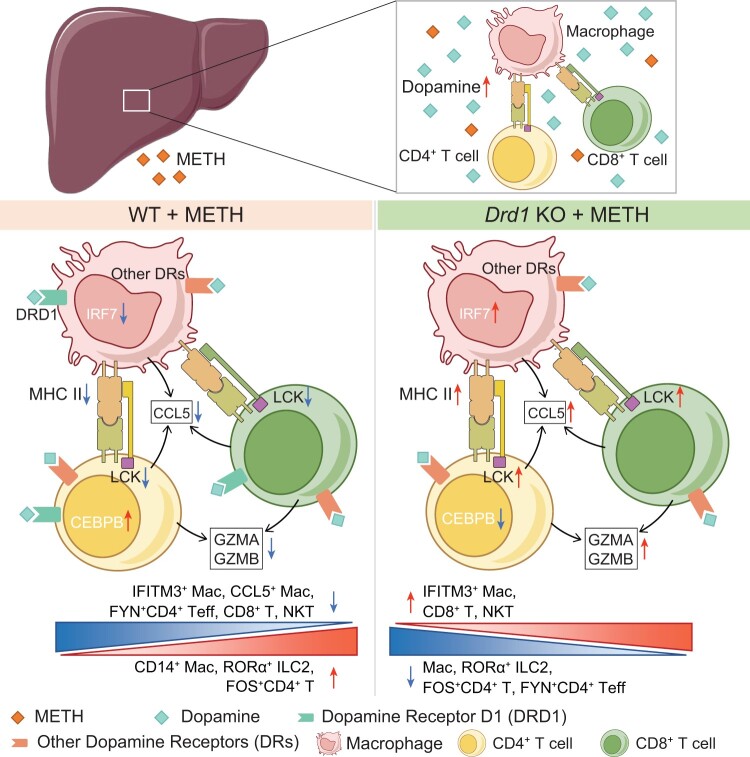

## Introduction

Methamphetamine (METH or MA; crystal methamphetamine is also known as ice) is a highly addictive and neurotoxic drug [1]. According to the World Drug Report 2023, approximately 36 million people worldwide had used amphetamines in 2021, and METH is the most frequently abused drug in East and Southeast Asia [2]. METH causes severe societal problems and it is also harmful to health, damaging the central nervous system and immune system and leading to susceptibility to infectious diseases, such as viral hepatitis and human immunodeficiency virus (HIV) [3–6]. Despite the fact that there have been an increasing number of relevant studies, the exact mechanism underlying the effect of METH on immune systems remains unclear.

Chronic METH use disrupts immune cell balance [4,7,8], affecting signaling pathways and causing damage to cells, such as splenic dendritic cells and T cells [4]. It also influences various homeostatic pathways, such as chemokine receptors and intracellular calcium levels, which are critical for immune responses [4]. The liver, the key digestive and frontline immune organ [6,9], experiences METH-induced toxicity [10], leading to hepatotoxicity, including hyperthermia [11], cell cycle arrest [12], deleterious inflammatory response [13], and reduced METH clearance [14]. However, there are limited studies on exploring changes in liver immune cells under chronic METH influence.

The dopamine (DA) system is crucial in METH-related disorders. DA facilitates neurotransmission through the activation of five DA receptors, which are grouped into two D1-like DA receptors [DA receptor D1 (DRD1) and DA receptor D5 (DRD5)] and three D2-like DA receptors [DA receptor D2 (DRD2), DA receptor D3 (DRD3), and DA receptor D4 (DRD4)] [15]. METH administration markedly increases DA levels in the nucleus accumbens [16]. Extracellular DA is crucial for activating DA receptors, and interacts with DRD1, DRD2, and DRD3 in the nucleus accumbens [15]. As a potent addictive psychostimulant through reward circuits, METH leads to both physical and psychological alterations [16,17], and its rewarding properties rely on the presence of DRD1 and DRD2 [18,19]. METH causes the extracellular release of DA and reduces DA reuptake through various mechanisms, such as blocking the role of DA transporter (DAT) [20] and increasing the expression of DRD1 [19]. A DRD1 antagonist has been reported to extinguish METH-induced conditioned place preference [21,22]. In addition to being a neurotransmitter, DA is an important regulator of immune function [23]. Immune cells produce DA, which can be used as an autocrine/paracrine mediator not only for immune cells themselves but also for adjacent cells [24]. DA receptors and other DA-related proteins have been detected in many immune cells, suggesting that DA is a crucial component in regulating immune function [25,26]. However, whether METH regulates liver immunity and the underlying mechanisms of METH in regulating immune function remain elusive. Therefore, investigating the regulatory mechanisms of METH through DA is a vital step in understanding its role in liver immunity.

Single-cell RNA sequencing (scRNA-seq) offers more powerful data for high-resolution analyses as compared with conventional bulk RNA sequencing (RNA-seq). Using scRNA-seq, cell subsets, subset functions, and cell–cell interactions in complex tissues can be identified at the single-cell transcriptomic level. METH has been reported to induce hepatotoxicity and to cause deleterious inflammatory responses [13,27]. However, scRNA-seq-based analysis to investigate the METH effect on liver immune cells has yet to be reported. In this study, we used scRNA-seq to explore METH-induced immunosuppression in the liver through DRD1. Examination of liver tissues from *Drd1*-knockout (KO) and wild-type (WT) mice chronically exposed to METH revealed changes in immune cell numbers and functions. This research may offer insights into treating METH addiction by understanding its impact on liver immunity.

## Results

### METH exposure and *Drd1* KO cause changes of immune cells in the mouse liver revealed by RNA-seq

To explore the METH effect on hepatic immune cells and the potential involvement of DRD1, we performed bulk RNA-seq by using mouse liver tissues from the WT + saline (WS), *Drd1* KO + saline (DS), WT + METH (WM), and *Drd1* KO + METH (DM) groups. Gene Ontology (GO) and Kyoto Encyclopedia of Genes and Genomes (KEGG) enrichment analyses of differentially expressed genes (DEGs) among the four groups showed that many immune-related pathways were affected by METH and *Drd1* KO, including response to interferon (IFN), defense response to virus, and regulation of innate immune response (Figure S1A; Tables S1 and S2). In addition, immune infiltration analysis demonstrated changes related to the proportion of some liver immune cells caused by METH exposure and *Drd1* KO. Flow cytometry analysis further supported that METH exposure and *Drd1* KO altered the proportion of certain cell types; for example, WM group exhibited increased CD4^+^ T cells and decreased CD8^+^ T cells, whereas DM group displayed a high proportion of CD8^+^ T cells and a trend of decreased macrophages (Macs) (Figure S1B and C). These results suggest that METH exposure affects the number and functions of liver immune cells under the involvement of DRD1 in the process.

### scRNA-seq data allow precise mapping of distinct immune cell populations

**Figure 1:**
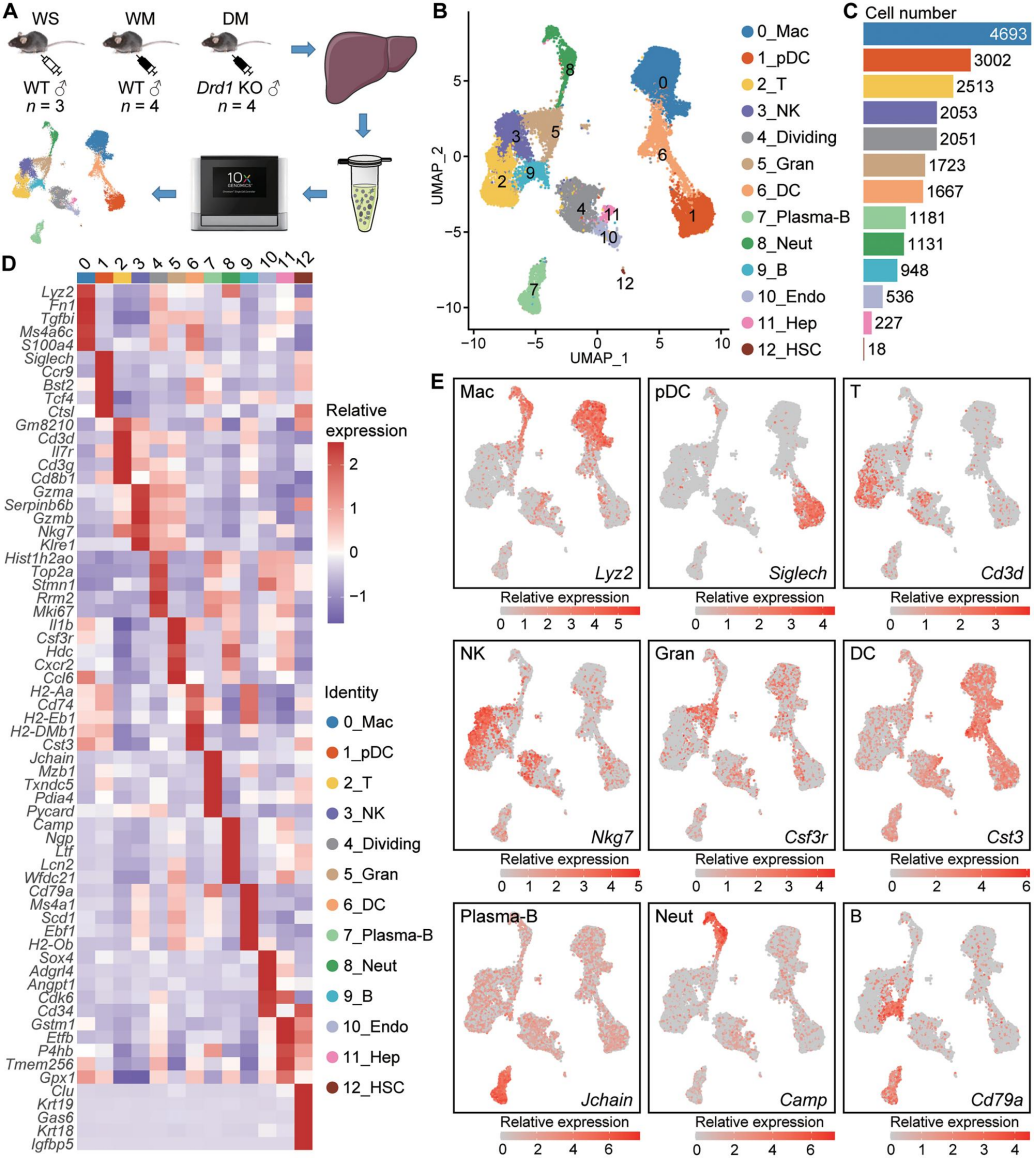
scRNA-seq reveals immune cell populations in mouse liver **A**. Schematic diagram for single-cell transcriptomic profiling of mouse NPCs. WS, WT + saline; WM, WT + METH; DM, *Drd1* KO + METH. **B**. and **C**. Overview of the 13 types of hepatic NPCs (B) and their counts (C). **D**. The signature genes for each cell type. **E**. The expression profiles of representative marker genes for the main immune cell types. NPC, nonparenchymal cell; WT, wild-type; *Drd1*, dopamine receptor D1; KO, knockout; METH, methamphetamine; UMAP, Uniform Manifold Approximation and Projection; Mac, macrophage; pDC, plasmacytoid DC; T, T cell; NK, natural killer cell; Diving, dividing cell; Gran, granulocyte; DC, dendritic cell; Plasma-B, plasma B cell; Neut, neutrophil; B, B cell; Endo, endothelial cell; Hep, hepatocyte; HSC, hepatic stellate cell; scRNA-seq, single-cell RNA sequencing.

After quality control and filtering, we obtained 21,743 transcriptomes at single-cell resolution from hepatic nonparenchymal cells (NPCs) of three mouse groups (WS group, *n* = 3; WM group, *n* = 4; DM group, *n* = 4) (**Figure 1**A). Based on the expression of canonical gene markers, we identified 13 distinct cell clusters with their unique markers (Figure 1B and C), nine of which are immune cells (Figure 1D; Table S3), including Mac (*Lyz2*^+^), plasmacytoid dendritic cell (pDC, *Siglech*^+^), T cell (T, *Cd3d*^+^), natural killer cell (NK, *Nkg7*^+^), granulocyte (Gran, *Csf3r*^+^), dendritic cell (DC, *Cst3*^+^), plasma B cell (Plasma-B, *Jchain*^+^), neutrophil (Neut, *Camp*^+^), and B cell (B, *Cd79a*^+^) (Figure 1E) [28,29]. A cluster identified based on highly-expressed *Mki67* (a marker gene for cell proliferation) [28,30] was named dividing cells (4_Dividing), which contains NK and T cells, as well as myeloid cells and hepatocytes (Figure 1D, Figure S2). These results indicate that the 4_Dividing cluster may be the progenitors of the aforementioned cells.

### METH induces immunosuppressive hepatic microenvironments partially through DRD1

**Figure 2:**
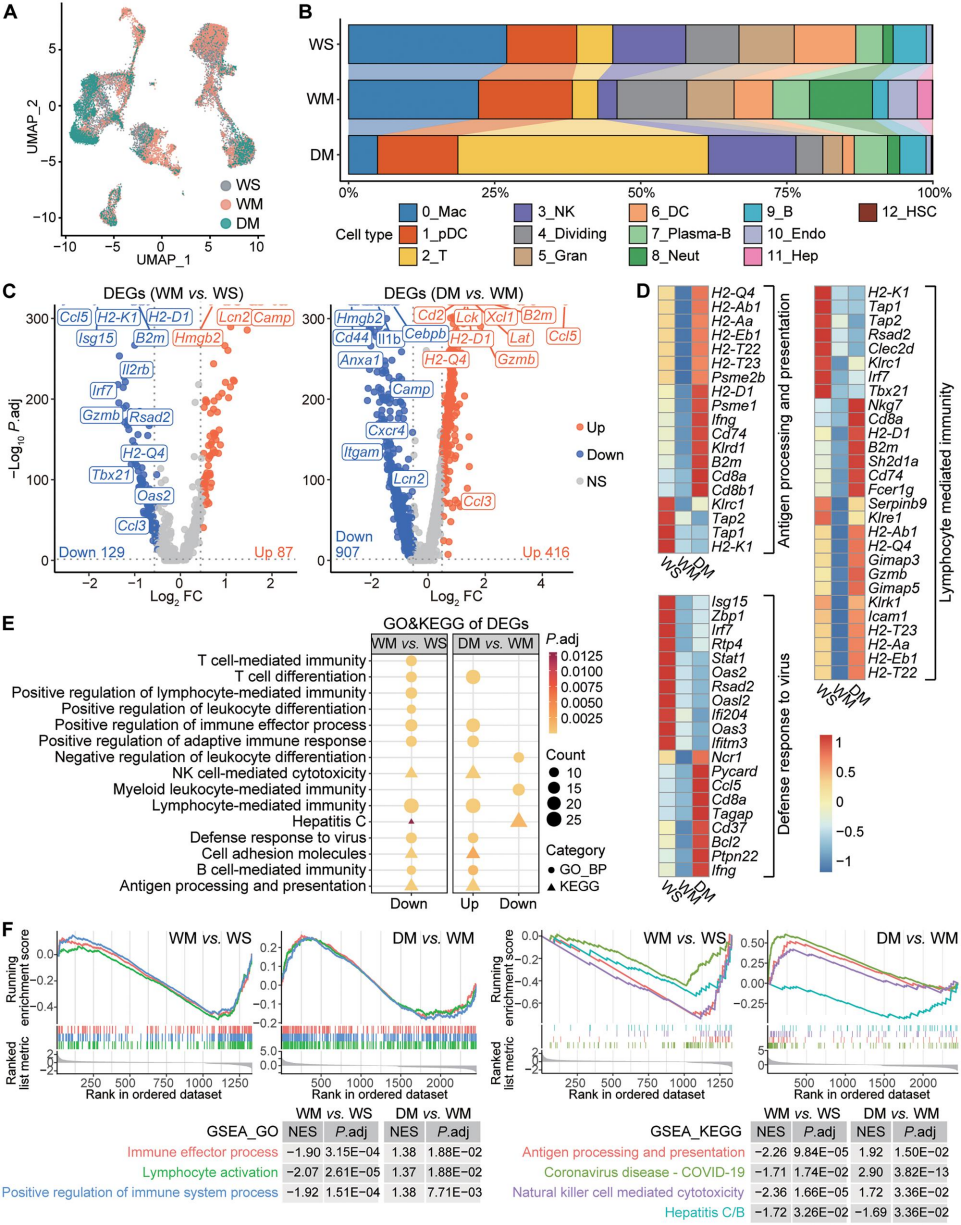
Comparative transcriptomic analyses reveal the METH-induced immunosuppressive hepatic microenvironments **A**. Cell populations from three groups of mice. **B**. Proportion of each cell cluster in three groups of mice. **C**. Volcano plots showing FCs of gene expression for down-regulated and up-regulated genes in comparisons of WM *vs*. WS and DM *vs*. WM. Red, blue, and gray dots represent the up-regulated (*P*.adj < 0.05 and log_2_ FC > 0.5), down-regulated (*P*.adj < 0.05 and log_2_ FC < −0.5), and non-significant (*P*.adj ≥ 0.05 or |log_2_ FC| ≤ 0.5) genes, respectively. DEGs with −log_10_*P*.adj value of infinity (*P*.adj is 0) are NOT fully shown in the plots (at the top of the plots), of which *Ccl5*, *Isg15*, *H2-K2*, *B2m*, *Hmgb2*, *Cd44*, *Il1b*, *Cebpb*, *Cd2*, *Lck*, *H2-D1*, *Xcl1*, *Lat*, and *Gzmb* are displayed with guide lines. **D**. Relative gene expression of representative immune pathways. **E**. GO and KEGG enrichment for the up-regulated and down-regulated genes in (C). **F**. GSEA enrichment of all DEGs (*P*.adj < 0.05). *P*.adj, adjusted *P* value; FC, fold change; NS, non-significant; GO, Gene Ontology; KEGG, Kyoto Encyclopedia of Genes and Genomes; GSEA, Gene Set Enrichment Analysis; DEG, differentially expressed gene; BP, biological process; NES, normalized enrichment score.

To investigate distinctive immune profiles for different groups, we analyzed the cell components of each group (**Figure 2**A and B; Table S4) and found that Macs were enriched in WS and WM, and Neut cells in WM. In the DM group, more than 50% of T cells were preferentially enriched, whereas the populations of Macs, Gran cells, and Neut cells decreased significantly, indicating that DRD1 is essential for the homeostasis of hepatic myeloid cells, while exhibiting an opposite effect on T cells.

DEG analysis revealed 87 up-regulated and 129 down-regulated genes between WM and WS, and 416 up-regulated and 907 down-regulated genes between DM and WM based on |log_2_ fold change (FC)| > 0.5 (Figure 2C; Table S5). The enrichment analysis of DEGs between WM and WS showed down-regulation of abundant immune-associated functional pathways, including antigen processing and presentation, lymphocyte-mediated immunity, positive regulation of immune effector process, NK cell-mediated immunity, defense response to virus, B cell-mediated immunity, and cell adhesion molecules. However, when comparing DM and WM, these pathways were up-regulated (Figure 2D–F; Tables S6 and S7). These findings indicate that chronic exposure to METH induces immunosuppressive hepatic microenvironments and that *Drd1* KO could partially reverse the effect. Among genes within these pathways, several DEGs were down-regulated in WM but up-regulated in DM, including histocompatibility-2 genes [encoding subunits of major histocompatibility complex I (MHC I) and MHC II], *Cd2*, *Icam1*, *Zap70*, *Il2rg*, *Gimap3/5* (encoding members of the GTPase of the immunity-associated protein family), *Serpinb9*, *Irf7*, *Gzmb*, *Ifng*, and *Cd74* (Figure 2D, Figure S3), suggesting that these genes may be suppressed by METH through DRD1.

### METH exposure increases immunosuppressive Macs but *Drd1* KO causes Mac loss

**Figure 3:**
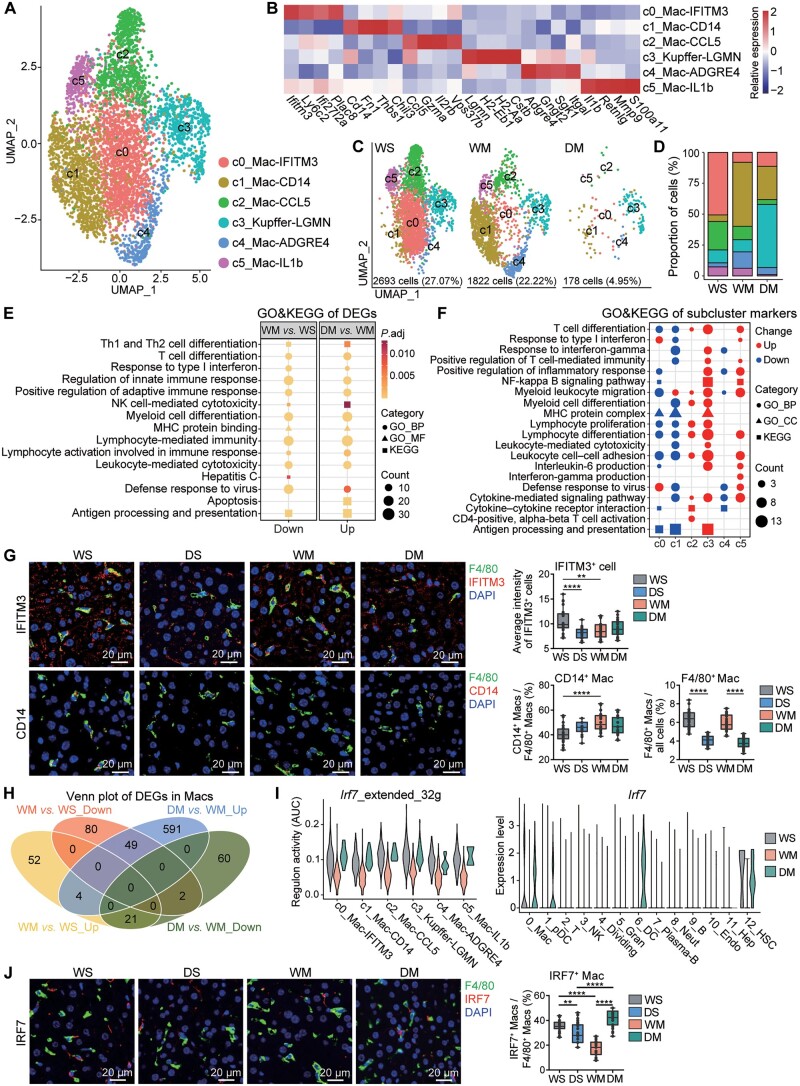
METH increase immunosuppressive Macs **A**. Six subclusters identified from the Macs. **B**. Heatmap showing the expression of marker genes in each Mac subcluster. **C**. and **D**. Group-wise cell populations (C) and proportions (D) of Macs. The legend is shared with (A). **E**. GO and KEGG enrichment of the up-regulated and down-regulated genes in Macs. **F**. GO and KEGG enrichment of the marker genes of the Mac subclusters. **G**. Left: representative double immunofluorescent images of mouse hepatic tissues (*n* = 3 for each group) with DAPI nuclear counterstain (×40). Scale bar, 20 μm. Right: average intensity of IFITM3^+^ cells, proportion of CD14^+^ Macs in all F4/80^+^ Macs, and proportion of F4/80^+^ Macs in all cells. **, *P* < 0.01; ****, *P* < 0.0001 (one-way ANOVA). **H**. The intersection of up-regulated and down-regulated genes in Macs. **I**. Regulon activity (AUC) of *Irf7* and its targets (left) and expression of *Irf7* in all cell types (right). Regulon activity is represented by the AUC of genes in regulon among all genes expressed in the cells. **J**. Left: representative double immunofluorescent images of mouse hepatic tissues (*n* = 3 for each group) with DAPI nuclear counterstain (×40). Scale bar, 20 μm. Right: proportion of IRF7^+^ Macs in all F4/80^+^ Macs. **, *P* < 0.01; ****, *P* < 0.0001 (one-way ANOVA). DS, *Drd1* KO + saline; MF, molecular function; CC, cellular component; DAPI, 4′,6-diamidino-2-phenylindole; AUC, area under the curve; ANOVA, analysis of variance.

As the largest immune cell population, 4693 Macs were identified (**Figure 3**A) and grouped into six subclusters, including c0_Mac-IFITM3, c1_Mac-CD14, c2_Mac-CCL5, c3_Kupffer-LGMN, c4_Mac-ADGRE4, and c5_Mac-IL1b (Figure 3A and B; Table S8). We observed significant differences in the composition of these subclusters between the three groups of mice. The proportions of c0_Mac-IFITM3 and c2_Mac-CCL5 in WS were significantly higher than those in WM and DM, the proportions of c1_Mac-CD14 and c4_Mac-ADGRE4 were higher in WM, and the proportion of c3_Kupffer-LGMN was higher in DM (Figure 3C and D).

Compared with WS, the proportion of Macs in WM decreased slightly. Unexpectedly, compared with WM, the proportion of Macs in DM decreased markedly and almost disappeared (Figure 3C). Furthermore, cell cycle scores showed G_2_M cell cycle arrest in WM and G_1_ cell cycle arrest in DM, suggesting suppression of proliferation in these two groups (Figure S4A). The DEG analysis showed that there were more down-regulated genes in WM as compared with WS, and these genes were enriched in many immune function pathways, such as antigen processing and presentation, defense response to virus, and T cell differentiation. Most of these pathways were reversed in DM, indicating that the impaired immunocompetence caused by METH exposure may occur via a mechanism involving DRD1 (Figure 3E, Figure S4B; Tables S9 and S10).

To identify functional changes in the shifts from c0_Mac-IFITM3 and c2_Mac-CCL5 in WS to c1_Mac-CD14 induced by METH exposure in WM, GO and KEGG enrichment analyses of the marker genes were performed (Figure 3F; Table S11). Compared with c0_Mac-IFITM3, which exhibited up-regulation of the defense response to virus pathway, c1_Mac-CD14 exhibited down-regulated myeloid leukocyte differentiation, leukocyte-mediated cytotoxicity, and defense response to virus pathways. Subcluster c2_Mac-CCL5 uniquely showed up-regulated lymphocyte proliferation and CD4^+^ αβT cell activation pathways. Subcluster c3_Kupffer-LGMN showed enhanced immune-related functions and may be functionally active. Although subcluster c4_Mac-ADGRE4 was only enriched in relatively fewer down-regulated pathways, it contained highly expressed genes such as *Adgre4*, *Gngt2*, and *Sgk1*, which may be related to intercellular information interactions. Subcluster c5_Mac-IL1b showed unique up-regulation of the *Ifng* production pathway, indicating the activation of its immune function. The proportions of c0_Mac-IFITM3 and c2_Mac-CCL5 were both decreased and shifted into c1_Mac-CD14 in WM, further demonstrating that METH exposure leads to suppression of immune functions (Figure 3D and G; considering that IFITM3 is a membrane protein and expressed in the majority of cells, we used the average fluorescence intensity to measure its expression). In DM, c3_Kupffer-LGMN was significantly increased, which may not fully compensate for the suppressed function of Macs because their number was markedly reduced.

The change in the proportion of c3_Kupffer-LGMN between WM and WS was similar to that of all Macs, but different in DM, in which the proportion of c3_Kupffer-LGMN was increased (Figures 2B and 3D). The results of DEG analysis as well as GO and KEGG enrichment analyses of c3_Kupffer-LGMN were slightly different from those analyzed in all Macs (Figure S4C–E; Tables S12 and S13). Pathways such as antigen processing and presentation, T cell-mediated immunity, response to type I IFN, and defense response to virus were down-regulated in WM but up-regulated in DM, indicating that METH exposure suppresses Kupffer immune function but *Drd1* KO partially reverses this suppression. However, some pathways, such as myeloid leukocyte migration and leukocyte chemotaxis, were up-regulated in the compared groups.

Through Venn diagram analysis (VDA), we identified some genes regulated by METH but showed reversed effect when *Drd1* was knocked out, including membrane protein gene *Ifitm3*, genes encoding MHC, and transcription factor gene *Irf7* (Figure 3H; **Table 1**, Table S14). An interaction network of these genes was generated based on the STRING database (Figure S4F). We further performed transcription factor analysis by SCENIC on 0_Mac, and found that the expression of *Irf7* and its regulatory network [regulon activity, represented by the area under the curve (AUC) of genes in regulon among all genes expressed in the cells] were down-regulated in immune-suppressed subclusters c1_Mac-CD14 and c4_Mac-IL1b, which consisted of most Macs in WM (Figure 3I; Table S15). Decreasing *Irf7*, which is a key transcriptional regulator of type I IFN-dependent immune responses, demonstrates the inhibitory effect of METH on Mac immune responses [31]. The expression changes of *Irf7* in the liver Macs were verified by tissue immunofluorescence staining (Figure 3J).

**Table 1:** Genes regulated by METH through DRD1

Cell type	Genes encoding membrane proteins/receptors	Transcription factor genes	Genes encoding effectors
Only in Mac	*Cd74*, *Cd83*, *Fcer1g*, *Fcgr1*, *Gngt1*, *H2-Aa*, *H2-Ab1*, *H2-Eb1*, *H2-T22*, *H2-T23*, *Ifitm3*, *Ly6c2*, *Ly6e*, *Ms4a4c*, *Ms4a6b*, *Rtp4*	*Atf3*, *Ddit3*, *Id2*, ***Irf7***	*Ccl3*, *Ccl4*, *Cst3*, *Fn1*, *Irf7*, *Isg15*, *Metrnl*, *Mmp9*, *Npc2*, *Zbp1*
Only in T cell	*Cd24a*, *Cd44*, *Clec12a*, *Pdia6*	***Cebpb***, ***Fosl2***, *Ltf*	*Camp*, *Il1b*, *Lcn2*, *Wfdc17*
Shared in Mac and T cell	*B2m*, *H2-D1*, *H2-Q4*, *Ly6a*, *Nkg7*	*Hmgb2*	*Ccl5*, *Gzma*, *Gzmb*, *Ngp*, *Retnlg*, *S100a8*, *S100a9*, *Tgfbi*, *Thbs1*, *Wfdc21*

*Note*: Genes were from intersection of DEGs of the WM *vs.* WS group and the DM *vs.* WM group. Genes with overlapping results of transcription factor analysis and DEG analysis were marked in bold. METH, methamphetamine; DRD1, dopamine receptor D1; Mac, macrophage; DEG, differentially expressed gene; WT, wild-type; KO, knockout; WS, WT + saline; WM, WT + METH; DM, *Drd1* KO + METH; DS, *Drd1* KO + saline.

### METH exposure induces T cell immunodeficiency but *Drd1* KO partially prevents this activity

**Figure 4:**
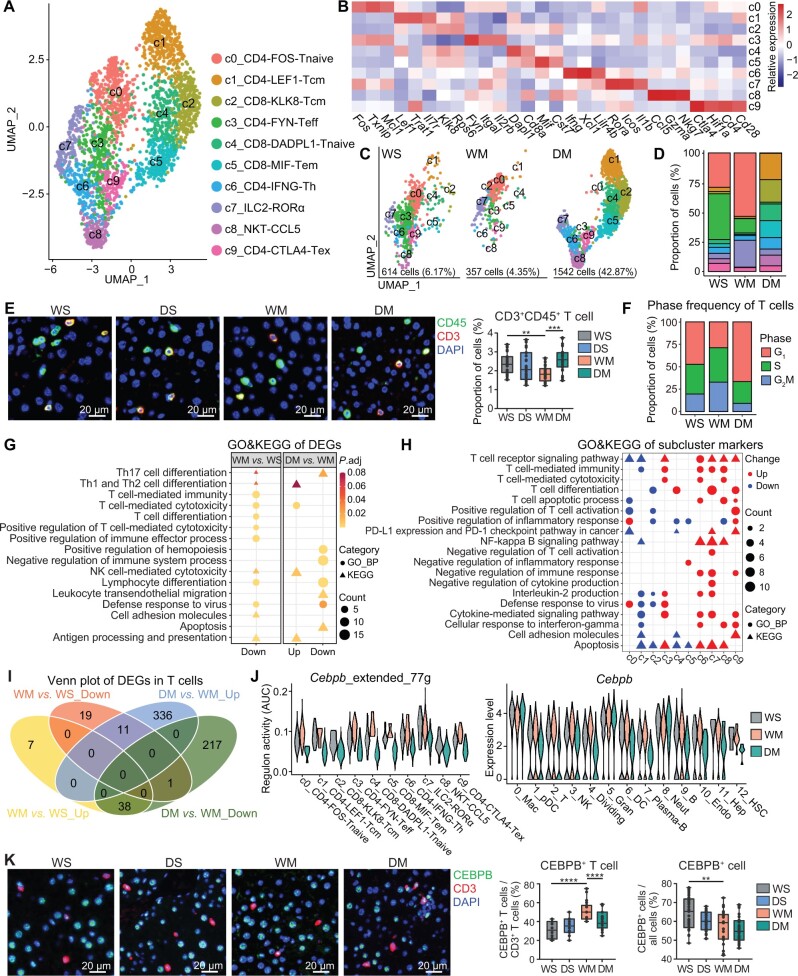
METH induces T cell immunodeficiency **A**. Ten subclusters identified from the T cells. **B**. Heatmap showing the expression of marker genes in each T cell subcluster. **C**. and **D**. Group-wise cell populations (C) and proportions (D) of T cells. The legend is shared with (A). **E**. Left: representative double immunofluorescent images of mouse hepatic tissues (*n* = 3 for each gourp) with DAPI nuclear counterstain (×40). Scale bar, 20 μm. Right: proportion of CD3^+^CD45^+^ T cells. *, *P* < 0.05; ****, *P* < 0.0001 (one-way ANOVA). **F**. Proportions of T cells at different cell cycle phases in the three groups. **G**. GO and KEGG enrichment of the up-regulated and down-regulated genes in T cells. **H**. GO and KEGG enrichment of the marker genes of the T cell subclusters. **I**. The intersection of up-regulated and down-regulated genes in T cells. **J**. Regulon activity (AUC) of *Cebpb* and its targets (left) and expression of *Cebpb* in all cell types (right). Regulon activity is represented by the AUC of genes in regulon among all genes expressed in the cells. **K**. Left: representative double immunofluorescent images of mouse hepatic tissues (*n* = 3 for each group) with DAPI nuclear counterstain (×40). Scale bar, 20 μm. Right: proportion of CEBPB^+^ T cells in all CD3^+^ T cells and proportion of CEBPB^+^ cells in all cells. **, *P* < 0.01; ****, *P* < 0.0001 (one-way ANOVA). G_1_, gap 1 phase; S, synthesis phase; G_2_M, gap 2/mitosis phase.

A total of 2513 T cells were identified according to traditional immunological surface markers (**Figure 4**A). These T cells were divided into ten subclusters, including four clusters for CD8^+^ T cells: c2_CD8-KLK8-Tcm (*Cd8*^+^*Ptprc*^−^*Ilr7*^+^), c4_CD8-DADPL1-Tnaive (*Cd8*^+^*Ptprc*^+^*Ccr7*^+^), c5_CD8-MIF-Tem (*Cd8*^+^*Ptprc*^−^*Prf1*^+^), and c8_NKT-CCL5 (*Cd8*^+^*NKg7*^+^); five clusters for CD4^+^ T cells: c0_CD4-FOS-Tnaive (*Cd4*^+^*Ptprc*^+^*Ccr7*^+^), c1_CD4-LEF1-Tcm (*Cd4*^+^*Ptprc*^−^*Ccr7*^+^), c3_CD4-FYN-Teff (*Cd4*^+^*Ptprc*^+^* Ccr7*^−^), c6_CD4-IFNG-Th (*Cd4*^+^*Ptprc^−^Ifng*^+^), and c9_CD4-CTLA4-Tex (*Cd4*^+^*Ctla4*^+^); and one cluster for innate lymphoid cells: c7_ILC2-RORα (*Cd4*^−^*Cd8*^−^*Rora*^+^) (Figure 4A and B; Table S16).

The number of T cells from DM significantly increased compared with WS and WM (Figures 2B, 4C, and 4D), which was verified by immunofluorescence of the liver tissue (Figure 4E). We observed that DM had the most G_1_ cells and the least G_2_M cells among the three groups (Figure 4F), and the apoptosis pathway was down-regulated concurrently (Figure 4G). Although the proportion of CD8^+^ T cells in DM increased significantly, most of them were naive and memory cells, except NKT. According to the enrichment results, *Drd1* KO eliminated some of the inhibition of METH on T cell differentiation and cytotoxicity. However, owing to the regulatory effect of DA on T cell [32,33], there may be some changes that we did not observe.

Compared with those in WS, the proportions of c0_CD4-FOS-Tnaive and c7_ILC2-RORα were increased and that of c3_CD4-FYN-Teff was decreased in WM, whereas c0_CD4-FOS-Tnaive and c3_CD4-FYN-Teff were almost absent in DM, with significant increases in c1_CD4-LEF1-Tcm, c2_CD8-KLK8-Tcm, c4_CD8-DADPL1-Tnaive, and c5_CD8-MIF-Tem, which were non-active naive or memory cells. GO and KEGG enrichment analyses as well as gene set variation analysis (GSVA) of the marker genes for each subcluster showed that the c0_CD4-FOS-Tnaive subcluster represented the naive cells, and c7_ILC2-RORα had the function of inhibiting T cell activation and immune response (Figure 4H, Figure S5A; Tables S17 and S18). These findings indicate that METH may inhibit T cell activation and differentiation. The high expression of *Icos* in ILC2 has been reported to induce an immunosuppressive microenvironment [34]. The proportions of subclusters c0_CD4-FOS-Tnaive and c7_ILC2-RORα decreased after *Drd1* KO. Subcluster c8_NKT-CCL5 NKT cells displayed strong immune activity, which almost disappeared in WM but was present in DM. Subcluster c9_CD4-CTLA4-Tex expressed high levels of exhaustion marker *Ctla4* and exhibited some fragile regulatory T cell (Treg) characteristics (high expression of *Hif1a* and loss of suppressive function) [35] (Figure S5B), but no increase in the proportion of this cell subcluster was detected in WM.

Compared with those in WS, T cells in WM exhibited down-regulation of differentiation, cytotoxicity, defense response to virus, and antigen processing and presentation pathways. In DM, the cytotoxicity and antigen processing and presentation pathways were up-regulated, but the defense response to virus pathway was still down-regulated (Figure 4G, Figure S5C; Tables S19 and S20).

Genes regulated by METH and reversely regulated by *Drd1* KO were also found in T cells by VDA (Figure 4I; Table 1), including transcription factor gene *Cebpb* and effector (secreted protein)-coding gene *Ccl5*. An interaction network of the genes regulated by METH via DRD1 in T cells, based on the STRING database, was generated (Figure S5D; Table S21). The transcription factor analysis on T cells demonstrated a higher expression level of *Cebpb* and its regulatory network in WM (mainly consisting of c0_CD4-FOS-Tnaive and c7_ILC2-RORα subclusters), with a lower expression level in the DM group (Figure 4J; Table S22). *Cebpb*, an inhibitor of T cell proliferation, is involved in many immunological processes in other immune cells [36–38]. The increase of CEBPB^+^ T cells and the decrease of CEBPB in WM when compared with WS may be related to the reduction of T cells and functional suppression of other immune cells. The expression changes of *Cebpb* in the liver T cells were verified by tissue immunofluorescence studies (Figure 4K).

### METH causes DRD1-related immunosuppression of NK and B cells

**Figure 5:**
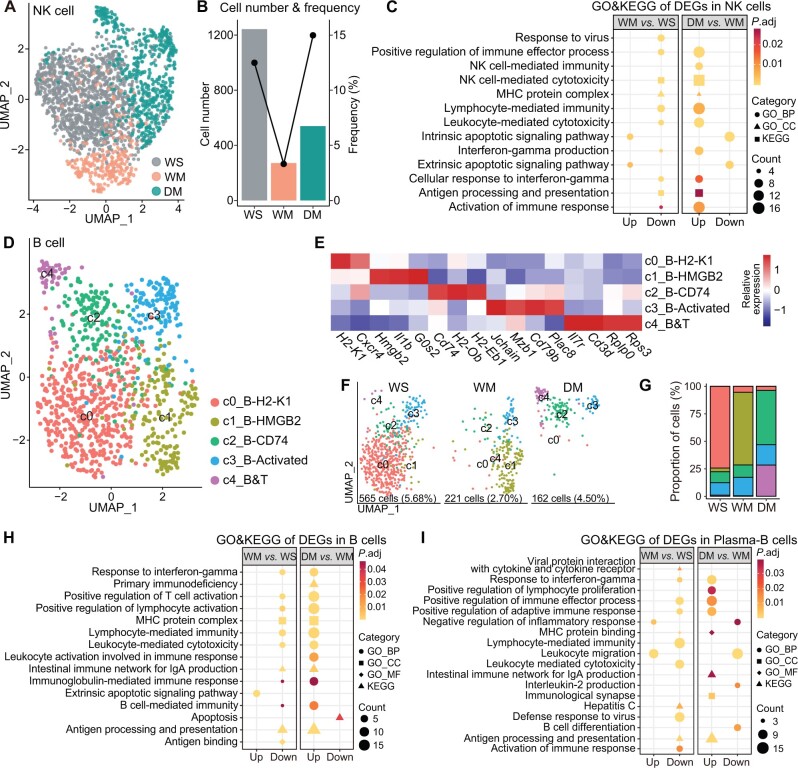
METH causes NK and B cells to exhibit DRD1-related immunosuppression **A**. UMAP visualization of NK cells from the three groups. **B**. Cell number and proportion of NK cells in the three groups. **C**. GO and KEGG enrichment of the up-regulated and down-regulated genes in NK cells. **D**. UMAP visualization of B cells showing a five-subcluster distribution. **E**. Heatmap showing the expression of marker genes in each B cell subcluster. **F**. and **G**. Group-wise cell populations (F) and proportions (G) of B cells. The legend is shared with (E). **H**. GO and KEGG enrichment of the up-regulated and down-regulated genes in B cells. **I**. GO and KEGG enrichment of the up-regulated and down-regulated genes in Plasma-B cells.

The changes in proportions and cell cycle scores of 2053 NK cells in the three groups (**Figure 5**A and B) were similar to those of T cells. Compared with those in WS, NK cells in WM showed down-regulation of the cytotoxicity, response to virus, and antigen processing and presentation pathways. In DM, these pathways were up-regulated, indicating that *Drd1* KO prevented the METH effect (Figure 5C; Tables S23 and S24). In contrast, the apoptosis-associated pathways were up-regulated in WM and down-regulated in DM.

A total of 948 B cells were further subdivided into five subclusters (Figure 5D; Table S25). As a subcluster of naive B cells, c0_B-H2-K1 was functionally inactive and highly expressed *Cxcr4*. Subcluster c1_B-HMGB2 displayed down-regulation of multiple immune functions and an increased proportion in WM. As activated B cells with high expression of *Jchain*, subcluster c3_B-Activated showed up-regulation of the B cell activation, differentiation, lymphocyte proliferation, activation of immune response, and response to IFN-γ pathways. Subcluster c4_B&T, which highly expressed *Cd3d*, showed up-regulation of the response to interleukin-4 and immunological synapse pathways and was a heterozygous cell population of activated B and T cells (Figure 5E–G, Figure S6; Table S26).

The proportion of B cells decreased in WM and increased in DM, with no significant difference in plasma-B cells among the three groups (Figures 2B and 5F). Compared with those in WS, B cells in WM showed down-regulation of the pathways of antigen processing and presentation, B cell-mediated immunity, and response to IFN-γ. Similar to T cells, B cells in DM showed recovery of some pathways that were down-regulated in WM, and a down-regulated apoptosis pathway (Figure 5G; Tables S27 and S28). For plasma-B cells, the changes in WM were similar to those observed in B cells. However, in DM, plasma-B cells showed distinct down-regulation of the B cell differentiation pathway and up-regulation of the positive regulation of lymphocyte proliferation and immunological synapse pathways (Figure 5H and I; Tables S29 and S30).

### METH inhibits cell crosstalk between Macs and T cells

CellChat analysis was performed to evaluate the probability of immune cell–cell communication by integrating gene expression data with prior knowledge of interactions between signaling ligands, receptors, and their cofactors. Since we observed that the functions of Kupffer cells and other five subclusters of Macs (hereafter referred to as ot-Macs) appeared to be different in the enrichment analyses, we analyzed these cells separately.

**Figure 6:**
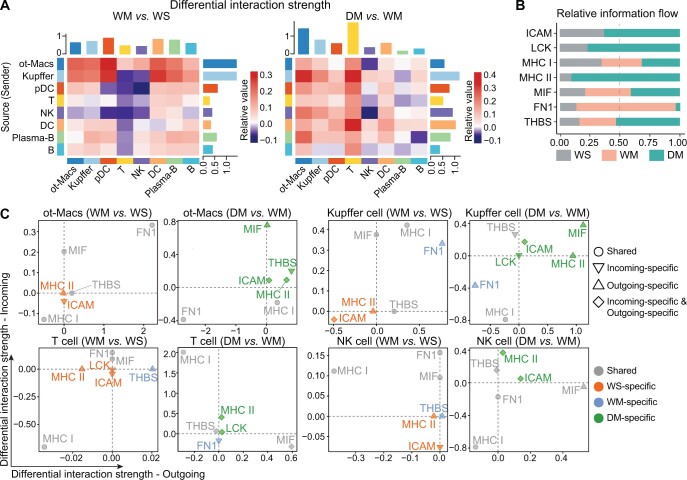
METH induces impaired crosstalk between immune cells **A**. Differential interaction strength of the three groups. Red (or blue) represents increased (or decreased) signaling in WM compared to WS (left) or in DM compared to WM (right). The top bar plot represents the sum of the values in each column of the heatmap (incoming signaling). The right bar plot represents the sum of the values in each row of the heatmap (outgoing signaling). **B**. Relative information flow of interested pathways in the three groups, which is defined by the sum of communication probability among all pairs of cell groups in the inferred network. **C**. Visualization of differential outgoing and incoming signaling changes in ot-Macs (subclusters c0, c1, c2, c4, and c5 of Macs), Kupffer cells (subcluster c3 of Macs), T cells, and NK cells from WM to WS as well as from DM to WM. Shape of dot: circle, outgoing or incoming signaling of the pathway was detected in both groups; inverted triangle, only incoming signaling of the pathway was detected in one group; triangle, only outgoing signaling of the pathway was detected in one group; diamond, both outgoing and incoming signaling of the pathway were detected in one group, or only one signaling of the pathway was detected in one group and the other signaling in another group. Color of point: gray, shared pathway (detected in two groups); red, WS-specific pathway (lost in WM); blue, WM-specific pathway (lost in WS); green, DM-specific pathway (lost in WM). ICAM, intercellular adhesion molecule; LCK, tyrosine–protein kinase LCK; MHC I, major histocompatibility complex I; MHC II, major histocompatibility complex II; MIF, macrophage migration inhibition factor; FN1, fibronectin 1; THBS, thrombospondin.

In the analysis of differential interaction strength, we found that, except for the increase in ot-Macs, METH resulted in decreased crosstalk of Kupffer cells, DCs, and all lymphocytes with T cells, and decreased crosstalk of Kupffer cells, pDCs, T cells, and NK cells with NK cells. However, *Drd1* KO led to the recovery of communication between T cells and other cells, but the crosstalk of NK cells with ot-Macs, T cells, and NK cells was further decreased, indicating that METH regulates ot-Macs, Kupffer cells, T cells, and NK cells differentially (**Figure 6**A).

In the analysis of the overall information flow of signal pathways, we found that WM appeared to have lost intercellular adhesion molecule (ICAM), tyrosine–protein kinase LCK, and MHC II [main pathway triggering CD4^+^ T cell receptor (TCR)] pathways, but exhibited a significant enhancement of the fibronectin 1 (FN1) pathway (Figure 6B; **Table 2**). Further analysis of these pathways of the main cell types that had changed (Figure 6C), we showed that: (1) the MHC II pathway disappeared in the WM, and the MHC I signal was markedly weakened, consistent with the down-regulation of the antigen processing and presentation pathway in most of the immune cells in this group; (2) in WM, the signal of the FN1 pathway in ot-Macs and Kupffer cells increased significantly, which may indicate an increase of immune infiltration or anti-inflammatory M2 [39]; (3) the ICAM signal disappeared in all cell types in WM, which may lead to the weakening of the inhibition of T cell pro-inflammatory factors [40]; (4) the LCK signal disappeared in T cells in WM, which may affect the triggering of TCR [41]; (5) the macrophage migration inhibition factor (MIF) signal increased in ot-Macs and Kupffer cells in DM. MIF is a pleiotropic cytokine with chemokine-like functions, which may be related to the decrease of Macs and the increase of B cells in DM [42,43]. The details of the cell–cell communication analysis are listed in Table S31.

**Table 2:** Summary of ligand–receptor pairs in the pathways

Pathway	Interaction (ligand–receptor)
ICAM	*Icam1*–*Itgal*, *Icam1*–*Spn*
LCK	*Lck*–(*Cd8a + Cd8b1*)
MHC I	*H2-T23*–*Cd8a*, *H2-T24*–*Cd8a*, *H2-D1*–*Cd8a*, *H2-K1*–*Cd8a*, *H2-Q4*–*Cd8a*, *H2-T23*–*Cd8b1*, *H2-T24*–*Cd8b1*, *H2-D1*–*Cd8b1*, *H2-K1*–*Cd8b1*, *H2-Q4*–*Cd8b1*, *H2-T10*–*Cd8b1*, *H2-T23*–(*Klrd1 + Klrc1*), *H2-M3*–*Cd8a*, *H2-Q10*–*Cd8a*, *H2-T22*–*Cd8a*, *H2-M3*–*Cd8b1*, *H2-Q10*–*Cd8b1*, *H2-T22*–*Cd8b1*
MHC II	*H2-Aa*–*Cd4*, *H2-Ab1*–*Cd4*, *H2-Eb1*–*Cd4*, *H2-Dma*–*Cd4*, *H2-Dmb1*–*Cd4*, *H2-Ob*–*Cd4*, *H2-Oa*–*Cd4*
MIF	*Mif*–(*Cd74 + Cxcr4*), *Mif*–(*Cd74 + Cd44*), *Mif*–(*Cd74 + Cxcr2*)
FN1	*Fn1*–(*Itga4 + Itgb1*), *Fn1*–(*Itga4 + Itgb7*), *Fn1*–*Cd44*, *Fn1*–*Sdc4*, *Fn1*–*Sdc1*
THBS	*Thbs1*–*Cd47*, *Thbs1*–*Sdc4*, *Thbs1*–*Cd36*

*Note*: ICAM, intercellular adhesion molecule; LCK, tyrosine–protein kinase LCK; MHC I, major histocompatibility complex I; MHC II, major histocompatibility complex II; MIF, migration inhibition factor; FN1, fibronectin 1; THBS, thrombospondin pathway.

## Discussion

In this study, by using scRNA-seq, we described changes of different liver-resident immune cells in response to METH and the possible role of DRD1 in this process. Our dataset provides new clues to possible therapeutics for METH-induced immune impairment.

Macs, as crucial elements of the innate immune system, can respond rapidly to various stimuli, such as infections of bacteria, viruses, or fungi, and other injuries. In this study, we found that chronic exposure to METH shifted the hepatic c0_Mac-IFITM3 (also named as *Ifitm3*^+^ Mac) and c2_Mac-CCL5 (also named as *Ccl5*^+^ Mac) to c1_Mac-CD14 (also named as *Cd14*^+^ Mac), with down-regulation of multiple functional immune pathways. This shift and down-regulation led to Mac suppression in WM, supporting that METH exposure leads to suppression of hepatic immune functions. Among affected immune factors, the decrease in *Ifitm3* and *Ccl5* and increase in *Cd14* (also named as monocyte differentiation antigen) in Macs (Figure 3G, Figure S4B) play a key role in the suppression of innate immunity by METH. *Ifitm3* restricts various viral infections and is closely related to the antiviral ability of cells [44,45]. CCL5 facilitates M1 polarization and impedes M2 polarization, and is associated with liver injury [46]. CD14, which is predominantly found in monocytes and Macs, is a marker of myeloid differentiation and inactivation. The surface expression of CD14 on monocytes is reduced after cell activation [47]. These results indicate that chronic exposure to METH impairs the antiviral or immune capacity of Macs. In addition, we found that Macs in DM almost disappeared, which was consistent with our immunofluorescence results (Figure 3G). Apart from the reason of G_1_ cell cycle arrest (Figure S4A), we believed that the loss of Macs was the result of *Drd1* KO rather than an effect of METH. The loss of Macs and its mechanism need to be confirmed and investigated further.

DA binds to D1-like receptors expressed by mouse Tregs and can inhibit the inhibitory activity of Tregs on Teff cells [48]. The concentration of DA also regulates the proliferation of T cells. At a physiological concentration range of 0.001–1 µM, DA usually suppresses activated Teff cells, leading to inhibition of their proliferation, cytokine secretion, and other processes and responses. However, at a much higher concentration of approximately 0.1–1 mM, DA can be toxic and kill T cells [32]. The administration of METH results in a marked increase in extracellular DA [20]. We speculate that changes in T cell numbers in WM and DM may be related to the increase in DA concentration due to METH and *Drd1* KO, respectively. In addition, the proportion of Macs in DS and DM decreased significantly, indicating that DRD1 is required for hepatic Mac homeostasis, but further research is needed to confirm this theory.

In our study, chronic exposure to METH inhibits the functions or proportions of most liver immune cells, leading to the down-regulation of some immune-related pathways. Some of these pathways are restored in the *Drd1* KO group (DM), indicating the involvement of DRD1 in the suppressive effects of METH. Among these pathways, the antigen processing and presentation pathway appears to be the most significant (Figures 3G, 4E, 5C, and 5G), especially in the communication between T cells and Macs or other antigen-presenting cells through MHC II, LCK, and ICAM, as well as in the communication between T cells and NK cells through LCK. These findings demonstrate that the structure and the function of DRD1 are associated with MHC II, LCK, and ICAM signaling pathways (Figure 6C; Table S31). We speculate that the main mode of METH affecting liver immunity may be via the DRD1–MHC II/DRD1–LCK relationship. The mechanism by which METH functions in immune-related pathways dependent or independent of DRD1 needs to be explored further.

Lymphocytes, especially T cells, are the main cells involved in adaptive immunity against viruses and other infections. METH is likely to cause disorders of other immune organs in addition to the liver [5], as shown for opioid addiction in previous studies. Opioid-associated blood exhibits an abnormal distribution of immune cells characterized by significant expansion of fragile Tregs, and shows enhanced Treg-derived IFN-γ expression [49]. In our study, there was a similar *Foxp3^+^* subcluster (c9_CD4-CTLA4-Tex) that lost its inhibitory function (Figure 4B). However, this subcluster had high expression of *Hif1a* but did not express IFN-γ (Figure S5B). Furthermore, our results showed that METH exposure decreased the ratio of CD8^+^ T cells in the liver, consistent with the results in peripheral blood [50], and inhibited a series of functions such as cell differentiation, proliferation, and cytotoxicity (Figure 4D and G). This discrepancy between our findings and those from studies on opioids may be related to the different effects and mechanisms of opioids and METH. Opioids are central nervous system inhibitors that act through opioid receptors, whereas METH is a central nerve stimulant that increases extracellular DA levels through interactions with DAT [20] and decreases the DAT expression [50]. Both DA and DAT have critical immunoregulation effects [51]. The addiction-associated mechanisms of opioids and METH are similar (related to DA and reward circuits), but their effects on the immune system may be different.

In our study, we found that chronic exposure to METH shifted the hepatic immune active c4_CD4-FYN-Teff (also named as *Fyn*^+^*Cd4*^+^ Teff), CD8^+^ T, and NKT (c8_NKT-CCL5) to c0_CD4-FOS-Tnaive (also named as *Fos*^+^*Cd4*^+^ T) and c7_ILC2-RORα (also named as *Rora*^+^ ILC2), and inhibited the function of B and NK cells, leading to lymphocyte immune suppression. Our data reveal that METH may regulate various effectors via DRD1, in which granzymes A and B (GZMA and GZMB) play a key role (Figure S7). Cytotoxic T lymphocytes and NK cells use perforin to deliver serine protease granzymes into target cells to kill them [52]. The decrease of granzyme levels in WM in our study confirmed the decreased cellular immune capacity.

This study also found that METH increased the oxidative stress of liver immune cells. The enrichment analysis of DEGs across most cell types, such as Mac, T cell, NK cell, and B cell, showed up-regulation of pathways related to peroxidase activity and cellular response to oxidative stress in WM (Tables S6, S10, S20, S24, S28, and S30). Compared with that in WM, the cell death pathway in response to oxidative stress was down-regulated in DM. We selected the top 50 oxidative stress-related genes with the highest correlation scores from the literature [53], and analyzed their relative expression levels in our data. We found that these genes were mostly up-regulated in WM (Figure S8). The proportion of Neut cells in WM was significantly increased (Figure 2B), which is consistent with previous reports on METH-induced liver Neut infiltration [54], and such an increase may also be linked to elevated oxidative stress.

Our study has some limitations. First, hepatic NPCs were isolated predominantly, which restricts understanding of the crosstalk between liver parenchymal cells and immune cells. Second, although bulk RNA-seq revealed some effects of *Drd1* KO on liver immune function, DS was not included in the scRNA-seq experiments as we focused on METH, thus the effects of *Drd1* KO alone on hepatic immunity at the single-cell level are not known. Third, the alterations in the proportions of some cell types, such as Macs, T cells, and B cells, in scRNA-seq were not completely consistent with those observed in other experiments, such as immunofluorescence, flow cytometry, or immune infiltration analysis in bulk RNA-seq. This discrepancy may be due to differences between messenger RNA (mRNA) and protein or differences in the technologies themselves. In this study, we used flow cytometry and scRNA-seq to quantify cells based on the recognition of cell surface protein markers by using specific antibodies and by analyzing mRNA expression, respectively. We also conducted the immune infiltration analysis to estimate the abundance of immune cells based on cell type-associated mRNAs. The levels of mRNA expression and protein synthesis differ inherently over time and are time-sensitive. As a result, there should be inconsistencies depending on mRNA and surface protein expression levels at the same or similar time points. Although scRNA-seq is a highly effective method for cell type-based gene expression analysis and visualization of such expression landscapes specific to different cell types, the interpretation of its data should be more careful, due to technical constraints such as experimental procedures and algorithms used for data analysis, especially when data across different studies are used.

Potential actions for future studies include: (1) clarifying the crosstalk between liver parenchymal cells and immune cells; (2) validating key findings in human samples (METH addict *vs*. healthy subject); (3) using diverse addiction models to study liver immunity; and (4) stratifying demographic parameters in METH chronic exposure studies, such as gender, age, and symptom severity.

In conclusion, we examined the effects of chronic METH exposure on hepatic immune cells and investigated whether DRD1 was involved based on the scRNA-seq data from two mouse models, METH-exposed and *Drd1*-KO mice. The chronic METH exposure shifted the immune cells from *Ifitm3*^+^ Mac and *Ccl5*^+ ^Mac to *Cd14*^+^ Mac as well as from *Fyn*^+^*Cd4*^+^ Teff, *Cd8*^+^ T, and NKT to *Fos*^+^*Cd4*^+ ^T and *Rora*^+^ ILC2, coupled with suppression of multiple functional immune pathways, and these effects were partially prevented by *Drd1* KO. Based on our findings, we propose that METH may suppress hepatic Macs through DA–DRD1/MHC II–IRF7–IFN/CCL5/others effectors and suppress T cells through DA–DRD1/MHC II–CEBPB–CCL5/others effectors.

## Materials and methods

### METH chronically exposed mouse model

The experimental animals were provided by Prof. Ming Xu (University of Chicago, USA). Genotypes of the *Drd1*-KO mice and their matched WT mice were determined by polymerase chain reaction (PCR) using gene-specific primers as previously reported [55]. All mice were housed in a pathogen-free animal facility at Xi’an Jiaotong University, and the temperature and humidity of the room were controlled. Mice were group-housed in a 12-h light/12-h dark cycle with free access to food and water.

METH chronically exposed mouse model was modified based on a previous study [56]. Briefly, METH was dissolved in sterile 0.9% physiological saline. Male 8-week-old WT (W set) and *Drd1*-KO mice (D set) were randomly divided into four groups (two groups per set, *n* = 8) and received intraperitoneal injections of saline (WS and DS) or METH (WM and DM) for 2 weeks. During the initial 7 days, the mice received daily injections of 1 mg/kg of body weight, and for the subsequent 7 days, the dose was increased to 2 mg/kg of body weight. The mice were sacrificed 24 h after the last injection under deep anesthesia, and the livers were dissected and stored according to the requirements of subsequent experiments.

METH (purity of 99.1%, identified by the National Institute for Food and Drug Control, Guangzhou, China) was obtained from the National Institute for the Control of Pharmaceutical and Biological Products (Beijing, China).

### Bulk RNA-seq and data processing

The liver tissues were used for bulk RNA-seq as previously described [57]. Briefly, total RNA was extracted from liver samples, and a complementary DNA (cDNA) library was constructed using the MGIEasy RNA Library Prep Kit V2 (Catalog No. 1000005269, MGI, Shenzhen, China) (*n* = 6 per group). We utilized limma (v3.52.4), edgeR (v3.38.4), and DESeq2 (v1.36.0) to analyze DEGs [58–61], with the intersection as the final result. DEGs were identified based on the criteria of *P* < 0.05 and |log_2_ FC| > 1. DEG enrichment analysis was performed using clusterProfiler (v4.4.4) [62]. Immune infiltration analysis was performed using ImmuCellAI-mouse (v0.1.0) [63].

### Liver NPC isolation

Primary hepatic NPCs were isolated using the digestion solution PSCeasy dispase (Catalog No. CA3001500, Cellapy, Beijing, China) according to the manufacturer’s instructions, as previously described [64]. Briefly, the liver was perfused with perfusion solution [calcium-free Hanks with 0.5 M ethylene diamine tetraacetic acid (EDTA)] for 5 min, and then minced and digested in PSCeasy dispase at 37°C for 20 min. The disassociated cells were filtered through a 40-μm cell strainer (Catalog No. 352340, Falcon, One Riverfront Plaza, NY), and then the immune cells and liver cells were separated by centrifugation at 50 *g* and 4°C for 5 min. Erythrocytes were lysed with red blood cell lysis buffer (Catalog No. C3702, Beyotime, Shanghai, China) for 2 min and washed twice. The liver NPCs were filtered again to remove dead cells before being captured using a 10X Chromium Controller (GCG-SR-1, 10X Genomics, Pleasanton, CA).

### Flow cytometry

The liver cells were collected by gently mashing liver tissue through a 45-μm cell strainer without liver perfusion. Subsequently, the cells and cell debris were separated by Percoll (Catalog No. 17089101, Cytiva, Uppsala, Sweden). Erythrocytes were lysed as described above. Flow cytometry was used for cell type analysis of NPCs as previously described [65]. Briefly, the isolated cells were centrifuged at 350 *g* for 5 min at 4°C, and then washed and re-suspended in cold phosphate-buffered saline, followed by incubation with anti-CD16/CD32 antibody (Catalog No. 101301, BioLegend, San Diego, CA) to block Fc receptors. Fluorochrome-conjugated antibodies, APC-conjugated anti-CD11b (Catalog No. 101211, BioLegend), brilliant violet 421-conjugated anti-F4/80 (Catalog No. 123131, BioLegend), APC/Cyanine7-conjugated anti-CD8a (Catalog No. 100713, BioLegend), PE-conjugated anti-CD4 (Catalog No. 100407, BioLegend), and Alexa Fluor 488-conjugated anti-CD3 (Catalog No. 100212, BioLegend), were used to distinguish cell types. Macs were gated as CD11b^+^F4/80^+^; CD4^+^ T cells were gated as CD3^+^CD4^+^CD8^−^; and CD8^+^ T cells were gated as CD3^+^CD8^+^CD4^−^. Samples were analyzed using BD FACSAria Fusion Flow Cytometer (FACSAria Fusion, BD, CA). Data were analyzed using FlowJo software (https://www.flowjo.com/).

### scRNA-seq and data processing

We used Cell Ranger (v2.0.1; 10X Genomics) to pool and process the raw RNA-seq data. All reads were aligned to the mouse transcriptome reference genome (*Mus*. *musculus*, GRCm38.p5) through the Cell Ranger count pipeline. After data aggregation, we performed all filtering, normalization, and scaling of data using Seurat (v4.1.0) [66,67]. Cells with fewer than 200 and more than 3000 detected genes, as well as cells with fewer than 600 unique molecular identifiers and more than 5% mitochondrial counts, were filtered out. Genes detected in fewer than 10 cells were removed. Gene count for each cell was normalized by total expression, multiplied by a scale factor of 10,000, and transformed into a log scale.

Principal component analysis based on the highly variable genes detected (dispersion of 2) was performed for dimensionality reduction, and the top 35 principal components were selected for further analysis. The clusters were visualized using Uniform Manifold Approximation and Projection (UMAP). The top expressed genes for each cluster and the DEGs between the three groups (WS, WM, and DM) were defined by the Seurat FindMarkers function with the Wilcoxon rank sum test. Cell type-specific gene signatures were determined from the overlaps of the top expressed genes of the cell clusters which were calculated in the previous step and canonical gene markers. DEGs were identified based on the criteria of adjusted *P* value (*P*.adj) < 0.05 and |log_2_ FC| > 0.5. Enrichment analyses of the DEGs, including GO, KEGG, and Gene Set Enrichment Analysis (GSEA), were performed by using clusterProfiler (v4.4.4) [62]. Intercellular crosstalk was inferred and analyzed using CellChat (v1.6.1) [68], which predicts major signaling inputs and outputs for cells by integrating gene expression with prior knowledge of the interactions between signaling ligands, receptors, and their cofactors. Transcription factors and their target genes were reconstructed and termed “regulons” by SCENIC (v1.2.0) [69].

### Immunofluorescence

Liver tissues from the four groups, WS, DS, WM, and DM (*n* = 3 per group), were fixed *in situ* with 4% paraformaldehyde and embedded in paraffin. The methods for staining, scanning, and observation of sections are as previously described [64]. The primary antibodies used in the immunofluorescence analysis were anti-CD3 (Catalog No. GB13014, Servicebio, Wuhan, China), anti-CD45 (Catalog No. GB113886, Servicebio), anti-F4/80 (Catalog No. GB113373, Servicebio), anti-CD14 (Catalog No. GB11254, Servicebio), anti-IFITM3 (Catalog No. GB114087, Servicebio), anti-IRF7 (Catalog No.GB111169, Servicebio), and anti-CEBPB (Catalog No. ET1610-9, HUABIO, Hangzhou, China). All primary antibodies were rabbit anti-mouse, and the signals were detected with Alexa Fluor 488-conjugated (Catalog No. GB25303, Servicebio) or Cy3-conjugated (Catalog No. GB21303, Servicebio) goat anti-rabbit IgG. Sections were scanned with PANNORAMIC DESK (3D HISTECH, Budapest, Hungary). The browser software CaseViewer (v2.4) was used to acquire images (×40). Cell proportion refers to the ratio of positive cells *vs*. all cells with nuclei counterstained by 4′,6-diamidino-2-phenylindole (DAPI), all F4/80^+^ Macs, or CD3^+^ T cells in the image. GraphPad Prism (v9.0) was used to calculate the statistically significant differences in the immunofluorescence data using analysis of variance (ANOVA) at a 95% confidence level. *P* < 0.05 indicates a threshold of statistical significance.

## Ethical statement

The animal study was reviewed and approved by the Xi’an Jiaotong University Health Science Center, China (Approve No. 2019-172).

## Supplementary Material

qzae060_Supplementary_Data

## Data Availability

The raw sequencing data and processed data in this study have been deposited in Gene Expression Omnibus (GEO: GSE247843). The raw sequencing data and processed data have also been deposited in the BioProject at the National Genomics Data Center (NGDC), Beijing Institute of Genomics (BIG), Chinese Academy of Sciences (CAS) / China National Center for Bioinformation (CNCB) (BioProject: PRJCA026681), and are publicly accessible at https://ngdc.cncb.ac.cn/bioproject/. The raw sequencing data and processed data have also been deposited in the Genome Sequence Archive and the Open Archive for Miscellaneous Data [70] at the NGDC, BIG, CAS / CNCB (GSA: CRA016806; OMIX: OMIX006594), and are publicly accessible at https://ngdc.cncb.ac.cn/gsa/ and https://ngdc.cncb.ac.cn/omix/, respectively.

## Code availability

The R codes used to perform analyses are available at GitHub (https://github.com/XiaomingLi-xjtu/METH-DRD1-Lv) and BioCode (https://ngdc.cncb.ac.cn/biocode/tools/7506).

## CRediT author statement

**Jin-Ting Zhou:** Investigation, Data curation, Formal analysis, Visualization, Writing – original draft, Writing – review & editing. **Yungang Xu:** Formal analysis, Supervision, Writing – review & editing. **Xiao-Huan Liu:** Investigation, Formal analysis. **Cheng Cheng:** Funding acquisition, Investigation. **Jing-Na Fan:** Investigation. **Xiaoming Li:** Funding acquisition, Methodology, Supervision, Writing – review & editing. **Jun Yu:** Writing – review & editing. **Shengbin Li:** Conceptualization, Resources, Supervision, Writing – review & editing. All authors have read and approved the final manuscript.

## References

[1] Panenka WJ , Procyshyn RM, Lecomte T, MacEwan GW, Flynn SW, Honer WG, et al Methamphetamine use: a comprehensive review of molecular, preclinical and clinical findings. Drug Alcohol Depend 2013;129:167–79. 23273775 10.1016/j.drugalcdep.2012.11.016

[2] United Nations Office on Drugs and Crime. World drug report 2023. [Internet]. Vienna, Austria: United Nations Office on Drugs and Crime; 2023, https://www.unodc.org/unodc/en/data-and-analysis/world-drug-report-2023.html.

[3] Jones CM , Houry D, Han B, Baldwin G, Vivolo-Kantor A, Compton WM. Methamphetamine use in the United States: epidemiological update and implications for prevention, treatment, and harm reduction. Ann N Y Acad Sci 2022;1508:3–22. 34561865 10.1111/nyas.14688 PMC9097961

[4] Papageorgiou M , Raza A, Fraser S, Nurgali K, Apostolopoulos V. Methamphetamine and its immune-modulating effects. Maturitas 2019;121:13–21. 30704560 10.1016/j.maturitas.2018.12.003

[5] Macur K , Ciborowski P. Immune system and methamphetamine: molecular basis of a relationship. Curr Neuropharmacol 2021;19:2067–76. 33913404 10.2174/1570159X19666210428121632 PMC9185774

[6] Li Y , Li S, Xia Y, Li X, Chen T, Yan J, et al Alteration of liver immunity by increasing inflammatory response during co-administration of methamphetamine and atazanavir. Immunopharmacol Immunotoxicol 2020;42:237–45. 32249638 10.1080/08923973.2020.1745829

[7] Potula R , Haldar B, Cenna JM, Sriram U, Fan S. Methamphetamine alters T cell cycle entry and progression: role in immune dysfunction. Cell Death Discov 2018;4:44. 10.1038/s41420-018-0045-6 PMC5859078 29581895

[8] Lawson KS , Prasad A, Groopman JE. Methamphetamine enhances HIV-1 replication in CD4^+^ T-cells via a novel IL-1β auto-regulatory loop. Front Immunol 2020;11:136. 32117283 10.3389/fimmu.2020.00136 PMC7025468

[9] Kubes P , Jenne C. Immune responses in the liver. Annu Rev Immunol 2018;36:247–77. 29328785 10.1146/annurev-immunol-051116-052415

[10] Blaker AL , Northrop NA, Yamamoto BK. Peripheral influences of methamphetamine neurotoxicity. In: Preedy VR, editors. Neuropathology of drug addictions and substance misuse. San Diego: Academic Press; 2016, p.309–19.

[11] Matsumoto RR , Seminerio MJ, Turner RC, Robson MJ, Nguyen L, Miller DB, et al Methamphetamine-induced toxicity: an updated review on issues related to hyperthermia. Pharmacol Ther 2014;144:28–40. 24836729 10.1016/j.pharmthera.2014.05.001 PMC4700537

[12] Wang Q , Wei LW, Xiao HQ, Xue Y, Du SH, Liu YG, et al Methamphetamine induces hepatotoxicity via inhibiting cell division, arresting cell cycle and activating apoptosis: *in vivo* and *in vitro* studies. Food Chem Toxicol 2017;105:61–72. 28341135 10.1016/j.fct.2017.03.030

[13] Si Z , Yang G, Wang X, Yu Z, Pang Q, Zhang S, et al An unconventional cancer-promoting function of methamphetamine in hepatocellular carcinoma. Life Sci Alliance 2023;6:e202201660. 36669783 10.26508/lsa.202201660 PMC9873983

[14] Hambuchen MD , Berquist MD, Simecka CM, McGill MR, Gunnell MG, Hendrickson HP, et al Effect of bile duct ligation-induced liver dysfunction on methamphetamine pharmacokinetics and locomotor activity in rats. J Pharm Pharm Sci 2019;22:301–12. 31329536 10.18433/jpps30471 PMC7458465

[15] Chen X , Xing J, Jiang L, Qian W, Wang Y, Sun H, et al Involvement of calcium/calmodulin-dependent protein kinase II in methamphetamine-induced neural damage. J Appl Toxicol 2016;36:1460–7. 26923100 10.1002/jat.3301

[16] Yorgason JT , Hedges DM, Obray JD, Jang EY, Bills KB, Woodbury M, et al Methamphetamine increases dopamine release in the nucleus accumbens through calcium-dependent processes. Psychopharmacology 2020;237:1317–30. 31965252 10.1007/s00213-020-05459-2 PMC7196509

[17] Hossain MK , Hassanzadeganroudsari M, Kypreos E, Feehan J, Apostolopoulos V. Immune to addiction: how immunotherapies can be used to combat methamphetamine addiction. Expert Rev Vaccines 2021;20:707–15. 33970739 10.1080/14760584.2021.1927725

[18] Abraham AD , Neve KA, Lattal KM. Effects of D1 receptor knockout on fear and reward learning. Neurobiol Learn Mem 2016;133:265–73. 27423521 10.1016/j.nlm.2016.07.010 PMC5001556

[19] Shen B , Zhang D, Zeng X, Guan L, Yang G, Liu L, et al Cannabidiol inhibits methamphetamine-induced dopamine release via modulation of the DRD1–MeCP2–BDNF–TrkB signaling pathway. Psychopharmacology 2022;239:1521–37. 34997862 10.1007/s00213-021-06051-y

[20] Sambo DO , Lebowitz JJ, Khoshbouei H. The sigma-1 receptor as a regulator of dopamine neurotransmission: a potential therapeutic target for methamphetamine addiction. Pharmacol Ther 2018;186:152–67. 29360540 10.1016/j.pharmthera.2018.01.009 PMC5962385

[21] Nouri K , Anooshe M, Karimi-Haghighi S, Mousavi Z, Haghparast A. Involvement of hippocampal D1-like dopamine receptors in the inhibitory effect of cannabidiol on acquisition and expression of methamphetamine-induced conditioned place preference. Neurochem Res 2021;46:2008–18. 33993443 10.1007/s11064-021-03350-w

[22] Saika F , Kiguchi N, Wakida N, Kobayashi D, Fukazawa Y, Matsuzaki S, et al Upregulation of CCL7 and CCL2 in reward system mediated through dopamine D1 receptor signaling underlies methamphetamine-induced place preference in mice. Neurosci Lett 2018;665:33–7. 29174638 10.1016/j.neulet.2017.11.042

[23] Kawano M , Takagi R, Saika K, Matsui M, Matsushita S. Dopamine regulates cytokine secretion during innate and adaptive immune responses. Int Immunol 2018;30:591–606. 30165447 10.1093/intimm/dxy057

[24] Nakano K , Higashi T, Takagi R, Hashimoto K, Tanaka Y, Matsushita S. Dopamine released by dendritic cells polarizes Th2 differentiation. Int Immunol 2009;21:645–54. 19332443 10.1093/intimm/dxp033

[25] Matt SM , Gaskill PJ. Where is dopamine and how do immune cells see it?: dopamine-mediated immune cell function in health and disease. J Neuroimmune Pharmacol 2020;15:114–64. 31077015 10.1007/s11481-019-09851-4 PMC6842680

[26] Arce-Sillas A , Sevilla-Reyes E, Alvarez-Luquin DD, Guevara-Salinas A, Boll MC, Perez-Correa CA, et al Expression of dopamine receptors in immune regulatory cells. Neuroimmunomodulation 2019;26:159–66. 31311029 10.1159/000501187

[27] Wang LB , Chen LJ, Wang Q, Xie XL. Silencing the *Tlr4* gene alleviates methamphetamine-induced hepatotoxicity by inhibiting lipopolysaccharide-mediated inflammation in mice. Int J Mol Sci 2022;23:6810. 35743253 10.3390/ijms23126810 PMC9224410

[28] Xiong X , Kuang H, Ansari S, Liu T, Gong J, Wang S, et al Landscape of intercellular crosstalk in healthy and NASH liver revealed by single-cell secretome gene analysis. Mol Cell 2019;75:644–60.e5. 31398325 10.1016/j.molcel.2019.07.028 PMC7262680

[29] Han X , Wang R, Zhou Y, Fei L, Sun H, Lai S, et al Mapping the mouse cell atlas by Microwell-seq. Cell 2018;172:1091–107.e17. 29474909 10.1016/j.cell.2018.02.001

[30] Hu C , Chu C, Liu L, Wang C, Jin S, Yang R, et al Dissecting the microenvironment around biosynthetic scaffolds in murine skin wound healing. Sci Adv 2021;7:eabf0787. 10.1126/sciadv.abf0787 PMC8153724 34039601

[31] Ren Y , Zhao Y, Lin D, Xu X, Zhu Q, Yao J, et al The type I interferon–IRF7 axis mediates transcriptional expression of *Usp25* gene. J Biol Chem 2016;291:13206–15. 27129230 10.1074/jbc.M116.718080 PMC4933234

[32] Levite M. Dopamine and T cells: dopamine receptors and potent effects on T cells, dopamine production in T cells, and abnormalities in the dopaminergic system in T cells in autoimmune, neurological and psychiatric diseases. Acta Physiol (Oxf) 2016;216:42–89. 25728499 10.1111/apha.12476

[33] Thomas Broome S , Louangaphay K, Keay KA, Leggio GM, Musumeci G, Castorina A. Dopamine: an immune transmitter. Neural Regen Res 2020;15:2173–85. 32594028 10.4103/1673-5374.284976 PMC7749467

[34] He Y , Luo J, Zhang G, Jin Y, Wang N, Lu J, et al Single-cell profiling of human CD127^+^ innate lymphoid cells reveals diverse immune phenotypes in hepatocellular carcinoma. Hepatology 2022;76:1013–29. 35243668 10.1002/hep.32444 PMC9790738

[35] Overacre-Delgoffe AE , Vignali DAA. Treg fragility: a prerequisite for effective antitumor immunity? Cancer Immunol Res 2018;6:882–7. 30068755 10.1158/2326-6066.CIR-18-0066 PMC6080214

[36] Berberich-Siebelt F , Berberich I, Andrulis M, Santner-Nanan B, Jha MK, Klein-Hessling S, et al SUMOylation interferes with CCAAT/enhancer-binding protein beta-mediated c-myc repression, but not IL-4 activation in T cells. J Immunol 2006;176:4843–51. 16585579 10.4049/jimmunol.176.8.4843

[37] Xu C , Xu J, Lu L, Tian W, Ma J, Wu M. Identification of key genes and novel immune infiltration-associated biomarkers of sepsis. Innate Immun 2020;26:666–82. 33100122 10.1177/1753425920966380 PMC7787554

[38] Yang J , Xu Y, Xie K, Gao L, Zhong W, Liu X. CEBPB is associated with active tumor immune environment and favorable prognosis of metastatic skin cutaneous melanoma. Front Immunol 2022;13:991797. 36353635 10.3389/fimmu.2022.991797 PMC9637891

[39] Wang H , Zhang J, Li H, Yu H, Chen S, Liu S, et al FN1 is a prognostic biomarker and correlated with immune infiltrates in gastric cancers. Front Oncol 2022;12:918719. 36081567 10.3389/fonc.2022.918719 PMC9445423

[40] Zheng S , Huang K, Xia W, Shi J, Liu Q, Zhang X, et al Mesenchymal stromal cells rapidly suppress TCR signaling-mediated cytokine transcription in activated T cells through the ICAM-1/CD43 interaction. Front Immunol 2021;12:609544. 33692786 10.3389/fimmu.2021.609544 PMC7937648

[41] Casas J , Brzostek J, Zarnitsyna VI, Hong JS, Wei Q, Hoerter JA, et al Ligand-engaged TCR is triggered by Lck not associated with CD8 coreceptor. Nat Commun 2014;5:5624. 25427562 10.1038/ncomms6624 PMC4248239

[42] Hughes CE , Nibbs RJB. A guide to chemokines and their receptors. FEBS J 2018;285:2944–71. 29637711 10.1111/febs.14466 PMC6120486

[43] Alampour-Rajabi S , El Bounkari O, Rot A, Muller-Newen G, Bachelerie F, Gawaz M, et al MIF interacts with CXCR7 to promote receptor internalization, ERK1/2 and ZAP-70 signaling, and lymphocyte chemotaxis. FASEB J 2015;29:4497–511. 26139098 10.1096/fj.15-273904

[44] Shi G , Kenney AD, Kudryashova E, Zani A, Zhang L, Lai KK, et al Opposing activities of IFITM proteins in SARS-CoV-2 infection. EMBO J 2021;40:e106501. 33270927 10.15252/embj.2020106501 PMC7744865

[45] Londrigan SL , Wakim LM, Smith J, Haverkate AJ, Brooks AG, Reading PC. IFITM3 and type I interferons are important for the control of influenza A virus replication in murine macrophages. Virology 2020;540:17–22. 31731106 10.1016/j.virol.2019.11.003

[46] Li M , Sun X, Zhao J, Xia L, Li J, Xu M, et al CCL5 deficiency promotes liver repair by improving inflammation resolution and liver regeneration through M2 macrophage polarization. Cell Mol Immunol 2020;17:753–64. 31481754 10.1038/s41423-019-0279-0 PMC7331700

[47] Shive CL , Jiang W, Anthony DD, Lederman MM. Soluble CD14 is a nonspecific marker of monocyte activation. AIDS 2015;29:1263–5. 26035325 10.1097/QAD.0000000000000735 PMC4452959

[48] Kipnis J , Cardon M, Avidan H, Lewitus GM, Mordechay S, Rolls A, et al Dopamine, through the extracellular signal-regulated kinase pathway, downregulates CD4^+^CD25^+^ regulatory T-cell activity: implications for neurodegeneration. J Neurosci 2004;24:6133–43. 15240805 10.1523/JNEUROSCI.0600-04.2004 PMC6729670

[49] Zhu Y , Yan P, Wang R, Lai J, Tang H, Xiao X, et al Opioid-induced fragile-like regulatory T cells contribute to withdrawal. Cell 2023;186:591–606.e23. 36669483 10.1016/j.cell.2022.12.030

[50] Gopinath A , Riaz T, Miller E, Phan L, Smith A, Syed O, et al Methamphetamine induces a low dopamine transporter expressing state without altering the total number of peripheral immune cells. Basic Clin Pharmacol Toxicol 2023;133:496–507. 36710070 10.1111/bcpt.13838 PMC10382601

[51] Gopinath A , Mackie PM, Phan LT, Mirabel R, Smith AR, Miller E, et al Who knew? Dopamine transporter activity is critical in innate and adaptive immune responses. Cells 2023;12:269. 36672204 10.3390/cells12020269 PMC9857305

[52] Zhou Z , He H, Wang K, Shi X, Wang Y, Su Y, et al Granzyme A from cytotoxic lymphocytes cleaves GSDMB to trigger pyroptosis in target cells. Science 2020;368:eaaz7548. 10.1126/science.aaz7548 32299851

[53] Wang H , Tian RF, Liang X, Fan J, Duan ZC, Fan XY, et al A four oxidative stress gene prognostic model and integrated immunity-analysis in pancreatic adenocarcinoma. Front Oncol 2023;12:1015042. 36713541 10.3389/fonc.2022.1015042 PMC9880292

[54] Peerzada H , Gandhi JA, Guimaraes AJ, Nosanchuk JD, Martinez LR. Methamphetamine administration modifies leukocyte proliferation and cytokine production in murine tissues. Immunobiology 2013;218:1063–8. 23518444 10.1016/j.imbio.2013.02.001 PMC5589440

[55] Xu M , Moratalla R, Gold LH, Hiroi N, Koob GF, Graybiel AM, et al Dopamine D1 receptor mutant mice are deficient in striatal expression of dynorphin and in dopamine-mediated behavioral responses. Cell 1994;79:729–42. 7954836 10.1016/0092-8674(94)90557-6

[56] Lai S , Wang J, Wang B, Wang R, Li G, Jia Y, et al Alterations in gut microbiota affect behavioral and inflammatory responses to methamphetamine in mice. Psychopharmacology 2022;239:1–16. 10.1007/s00213-022-06154-0 35503371

[57] Cheng C , Liu XH, He J, Gao J, Zhou JT, Fan JN, et al Apolipoprotein A4 restricts diet-induced hepatic steatosis via SREBF1-mediated lipogenesis and enhances IRS–PI3K–Akt signaling. Mol Nutr Food Res 2022;66:e2101034. 35909347 10.1002/mnfr.202101034

[58] Robinson MD , McCarthy DJ, Smyth GK. edgeR: a Bioconductor package for differential expression analysis of digital gene expression data. Bioinformatics 2010;26:139–40. 19910308 10.1093/bioinformatics/btp616 PMC2796818

[59] Love MI , Huber W, Anders S. Moderated estimation of fold change and dispersion for RNA-seq data with DESeq2. Genome Biol 2014;15:550. 25516281 10.1186/s13059-014-0550-8 PMC4302049

[60] Law CW , Chen Y, Shi W, Smyth GK. voom: precision weights unlock linear model analysis tools for RNA-seq read counts. Genome Biol 2014;15:R29. 24485249 10.1186/gb-2014-15-2-r29 PMC4053721

[61] Chen Y , Lun AT, Smyth GK. From reads to genes to pathways: differential expression analysis of RNA-seq experiments using Rsubread and the edgeR quasi-likelihood pipeline. F1000Res 2016;5:1438. 27508061 10.12688/f1000research.8987.1 PMC4934518

[62] Wu T , Hu E, Xu S, Chen M, Guo P, Dai Z, et al clusterProfiler 4.0: a universal enrichment tool for interpreting omics data. Innovation (Camb) 2021;2:100141. 34557778 10.1016/j.xinn.2021.100141 PMC8454663

[63] Miao YR , Zhang Q, Lei Q, Luo M, Xie GY, Wang H, et al ImmuCellAI: a unique method for comprehensive T-cell subsets abundance prediction and its application in cancer immunotherapy. Adv Sci (Weinh) 2020;7:1902880. 32274301 10.1002/advs.201902880 PMC7141005

[64] Liu XH , Zhou JT, Yan CX, Cheng C, Fan JN, Xu J, et al Single-cell RNA sequencing reveals a novel inhibitory effect of ApoA4 on NAFL mediated by liver-specific subsets of myeloid cells. Front Immunol 2022;13:1038401. 36426356 10.3389/fimmu.2022.1038401 PMC9678944

[65] Liu XH , Zhang Y, Chang L, Wei Y, Huang N, Zhou JT, et al Apolipoprotein A-IV reduced metabolic inflammation in white adipose tissue by inhibiting IKK and JNK signaling in adipocytes. Mol Cell Endocrinol 2023;559:111813. 36341820 10.1016/j.mce.2022.111813

[66] Stuart T , Butler A, Hoffman P, Hafemeister C, Papalexi E, Mauck WM 3rd, et al Comprehensive integration of single-cell data. Cell 2019;177:1888–902.e21. 31178118 10.1016/j.cell.2019.05.031 PMC6687398

[67] Hao Y , Hao S, Andersen-Nissen E, Mauck WM 3rd, Zheng S, Butler A, et al Integrated analysis of multimodal single-cell data. Cell 2021;184:3573–87.e29. 34062119 10.1016/j.cell.2021.04.048 PMC8238499

[68] Jin S , Guerrero-Juarez CF, Zhang L, Chang I, Ramos R, Kuan CH, et al Inference and analysis of cell–cell communication using CellChat. Nat Commun 2021;12:1088. 33597522 10.1038/s41467-021-21246-9 PMC7889871

[69] Van de Sande B , Flerin C, Davie K, De Waegeneer M, Hulselmans G, Aibar S, et al A scalable SCENIC workflow for single-cell gene regulatory network analysis. Nat Protoc 2020;15:2247–76. 32561888 10.1038/s41596-020-0336-2

[70] Chen T , Chen X, Zhang S, Zhu J, Tang B, Wang A, et al The Genome Sequence Archive Family: toward explosive data growth and diverse data types. Genomics Proteomics Bioinformatics 2021;19:578–83. 34400360 10.1016/j.gpb.2021.08.001 PMC9039563

