# Supplementary material for: Single-cell RNA-seq Reveals the Inhibitory Effect of Methamphetamine on Liver Immunity with the Involvement of Dopamine Receptor D1
Source: Genomics Proteomics Bioinformatics. 2024 Aug 28;22(4):qzae060. doi: 10.1093/gpbjnl/qzae060 (PMC11576359; doi:10.1093/gpbjnl/qzae060)
Supplement: qzae060_Supplementary_Data [file qzae060_supplementary_data.zip › supplementary material captions.docx]

# Supplementary material

**Figure S1 METH changed immune cells and immune-associated pathways in mice liver**

**A.** RNA-seq revealed changes in the immune-associated pathways in mice livers. WS, WT + saline (n = 6); WM, WT + METH (n = 6); DS, DRD1 KO + saline (n = 6); DM, DRD1 KO + METH (n = 6). **B.** Immune cell abundance scores estimated by ImmuCellAI-mouse based on RNA-seq data showed that the frequency of many immune cells may be changed after METH treatment. **C.** Frequency of different cell types in 4 groups by flow cytometry. Group information was the same as (A). *, *P* < 0.05; ***, *P* < 0.001.

**Figure S2 Dividing cell and its subclusters**

**A.** The cell cycle phases for all clusters. G_1_, gap 1 phase; S, synthesis phase; G_2_M, gap 2 / mitosis phase. **B.** and **C.** Group-wise cell populations (B) and proportions (C) of 4_Dividing cell. **D.** The expression profiles of *Mki67* in all cells and marker genes of the 4_Dividing cell population.

**Figure S3 Genes changed in the representative functional pathways**

Heatmap showing the relative gene expressions in the representative functional pathways.

**Figure S4 DEGs of** **macrophages and Kupffer cell**

**A.** Cell cycle phases of macrophages from 3 groups. **B.** Volcano plots showing FC of gene expression (log_2_ scale) for down-regulated and up-regulated genes in macrophages cells of WM *vs*. WS group and DM *vs*. WM group. Up-regulated genes (*P*.adj < 0.05 and log_2_FC > 0.5) are shown with red dots, down-regulated genes (*P*.adj < 0.05 and log_2_FC < −0.5) shown with blue dots, and insignificant genes (*P*.adj > 0.05 or log_2_FC < 0.5) shown with gray dots. **C.** Volcano plots showing FC of gene expression (log_2_ scale) for down-regulated and up-regulated genes in Kupffer cells of WM *vs*. WS group and DM *vs*. WM group. Up-regulated genes (*P*.adj < 0.05 and log_2_FC > 0.5) are shown with red dots, down-regulated genes (*P*.adj < 0.05 and log_2_FC < −0.5) shown with blue dots, and insignificant genes (*P*.adj > 0.05 or log_2_FC < 0.5) shown with gray dots. **D.** GO and KEGG enrichment of the up-regulated and down-regulated genes of Kupffer cells in (C). **E.** Heatmap showing the relative gene expression levels in the representative immune pathways of macrophages from 3 groups. **F.** STRING networks of the gene sets regulated by METH through DRD1 in macrophages.

**Figure S5 T subclusters function and DEGs**

**A.** GSVA of T-cell subclusters. **B.** The expressions of the feature genes from the c9_Cd4-Ctla4-Tex subcluster. **C.** Volcano plots showing FC of gene expression (log_2_ scale) for down-regulated and up-regulated genes in T cells of WM *vs*. WS group and DM *vs*. WM group. Up-regulated genes (*P*.adj < 0.05 and log_2_FC > 0.5) are shown with red dots, down-regulated genes (*P*.adj < 0.05 and log_2_FC < −0.5) shown with blue dots, and insignificant genes (*P*.adj > 0.05 or log_2_FC < 0.5) shown with gray dots. **D.** STRING networks of the gene sets regulated by METH through DRD1 in T cells. GSVA, gene set variation analysis.

**Figure S6 Enrichment of the marker genes of B subclusters**

GO and KEGG enrichment analysis of marker genes of five subclusters of B cells.

**Figure S7 The expression levels of *Gzma* and *Gzmb***

The expression levels of *Gzma* and *Gzmb* in all cell types from group WS, WM, and DM.

**Figure S8 The expression levels of top 50 of the reported oxidative stress-related genes**

**Table S1 Group-wise DEGs of RNA-seq**

**Table S2 Enrichment of DEGs of RNA-seq**

**Table S3 Top 50 markers genes of all cell type**

**Table S4 Cell number and frequency of all cell type from 3 group**

**Table S5 Group-wise DEGs of scRNA-seq (All cells)**

**Table S6 Enrichment of DEGs of scRNA-seq (All cells)**

**Table S7 GSEA enrichment of DEGs set of scRNA-seq (All cells)**

**Table S8 Top 30 markers genes of Mac subclusters**

**Table S9 Group-wise DEG of scRNA-seq (0_Mac except subcluster c3)**

**Table S10 Enrichment of DEGs (0_Mac except subcluster c3)**

**Table S11 Enrichment of subcluster markers (Mac subclusters)**

**Table S12 Group-wise DEG of scRNA-seq (c3_Kupffer-Lgmn)**

**Table S13 Enrichment of DEGs (Kupffer)**

**Table S14 Intersection of group-wise DEGs (Mac)**

**Table S15 Transcription factors analysis: regulon targets information (Mac)**

**Table S16 Top 30 markers genes of T subclusters**

**Table S17 Enrichment of subcluster markers (T subclusters)**

**Table S18 GSVA of T subclusters**

**Table S19 Group-wise DEGs of scRNA-seq (T cells)**

**Table S20 Enrichment of DEGs (T cells)**

**Table S21 Intersection of group-wise DEGs** **(T cells)**

**Table S22 Transcription factors analysis: regulon targets information (T cells)**

**Table S23 Group-wise DEGs of scRNA-seq (NK)**

**Table S24 Enrichment of DEGs (NK)**

**Table S25 Top 30 markers genes of B subclusters**

**Table S26 Enrichment of subcluster markers (B subclusters)**

**Table S27 Group-wise DEGs of scRNA-seq (B cells)**

**Table S28 Enrichment of DEGs (B cells)**

**Table S29 Group-wise DEG of scRNA-seq (Plasma-B)**

**Table S30 Enrichment of DEGs (Plasma-B)**

**Table S31 Cell Chat: all inferred cell−cell communications**
