# Supplementary figures and images for: Single-cell RNA-seq Reveals the Inhibitory Effect of Methamphetamine on Liver Immunity with the Involvement of Dopamine Receptor D1
Source: Genomics Proteomics Bioinformatics. 2024 Aug 28;22(4):qzae060. doi: 10.1093/gpbjnl/qzae060 (PMC11576359; doi:10.1093/gpbjnl/qzae060)

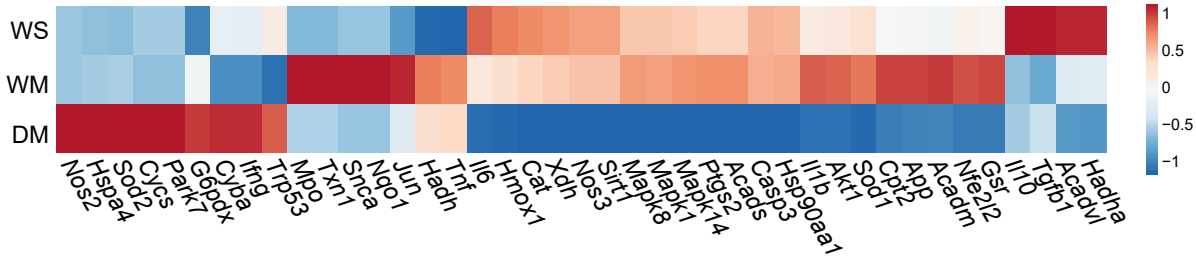

Supplement: qzae060_Supplementary_Data [file qzae060_supplementary_data.zip › Figure S8.pdf]

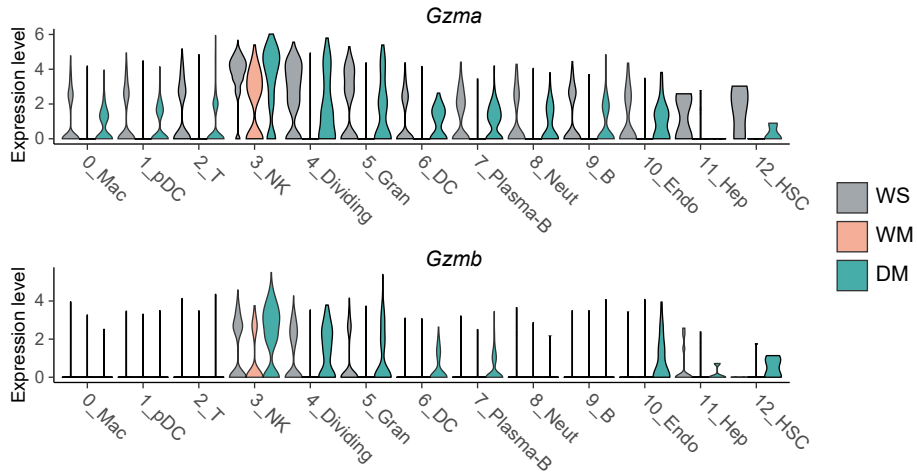

Supplement: qzae060_Supplementary_Data [file qzae060_supplementary_data.zip › Figure S7.pdf]

A

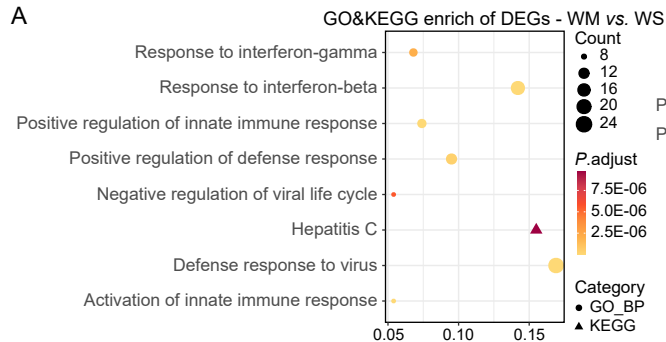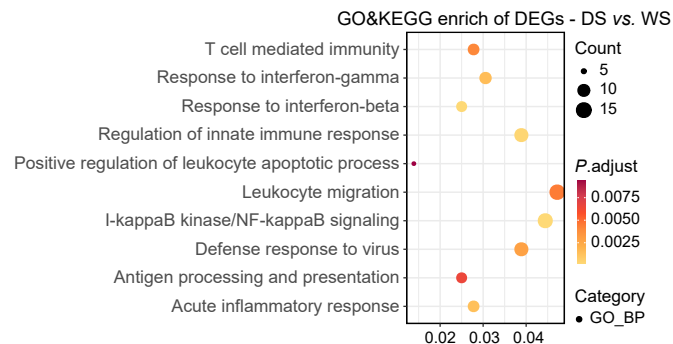

## B Immune infiltration analysis by ImmuCellAI-mouse

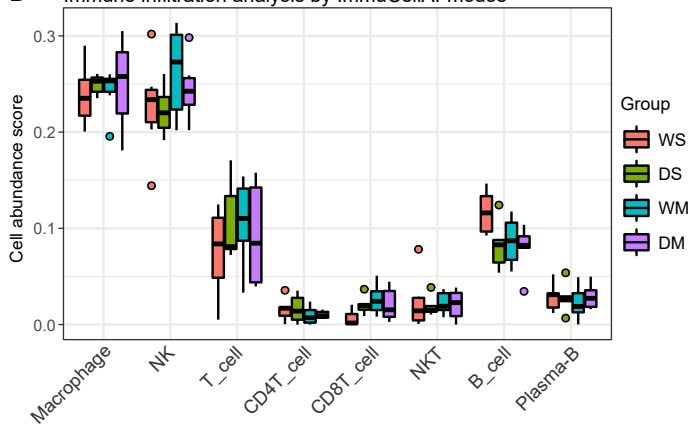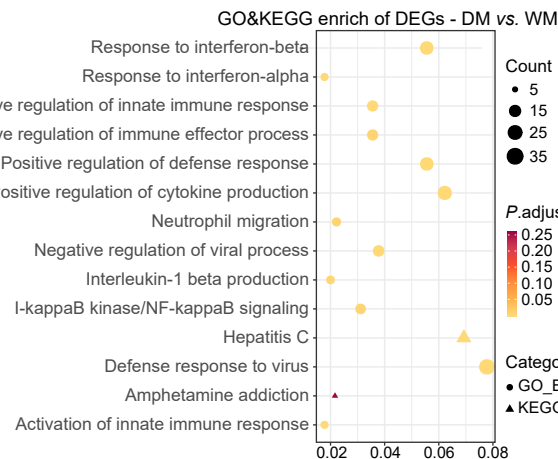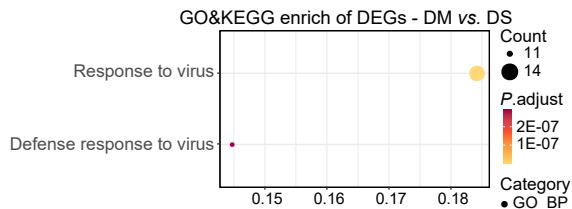

C

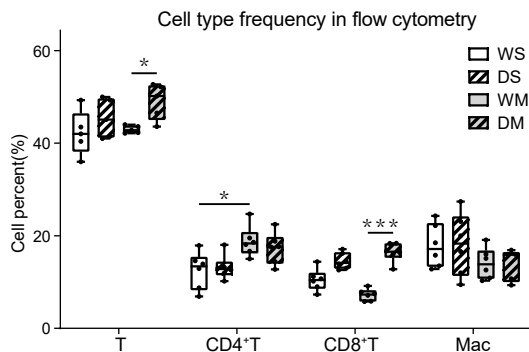

Supplement: qzae060_Supplementary_Data [file qzae060_supplementary_data.zip › Figure S1.pdf]

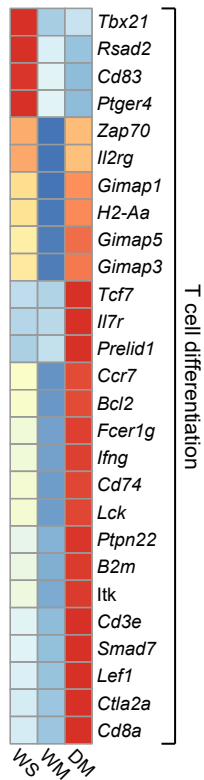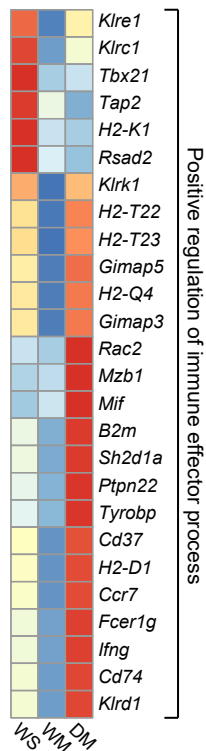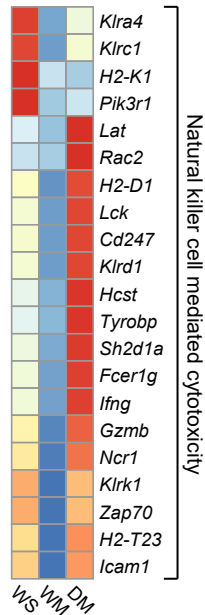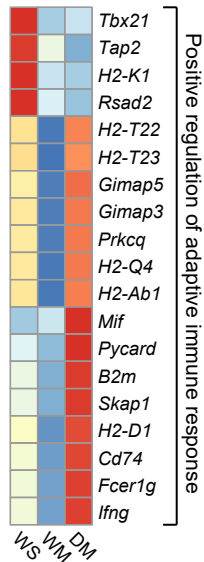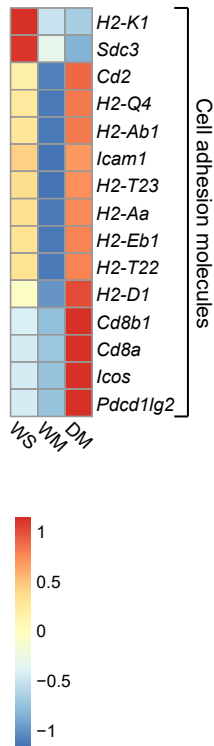

Supplement: qzae060_Supplementary_Data [file qzae060_supplementary_data.zip › Figure S3.pdf]

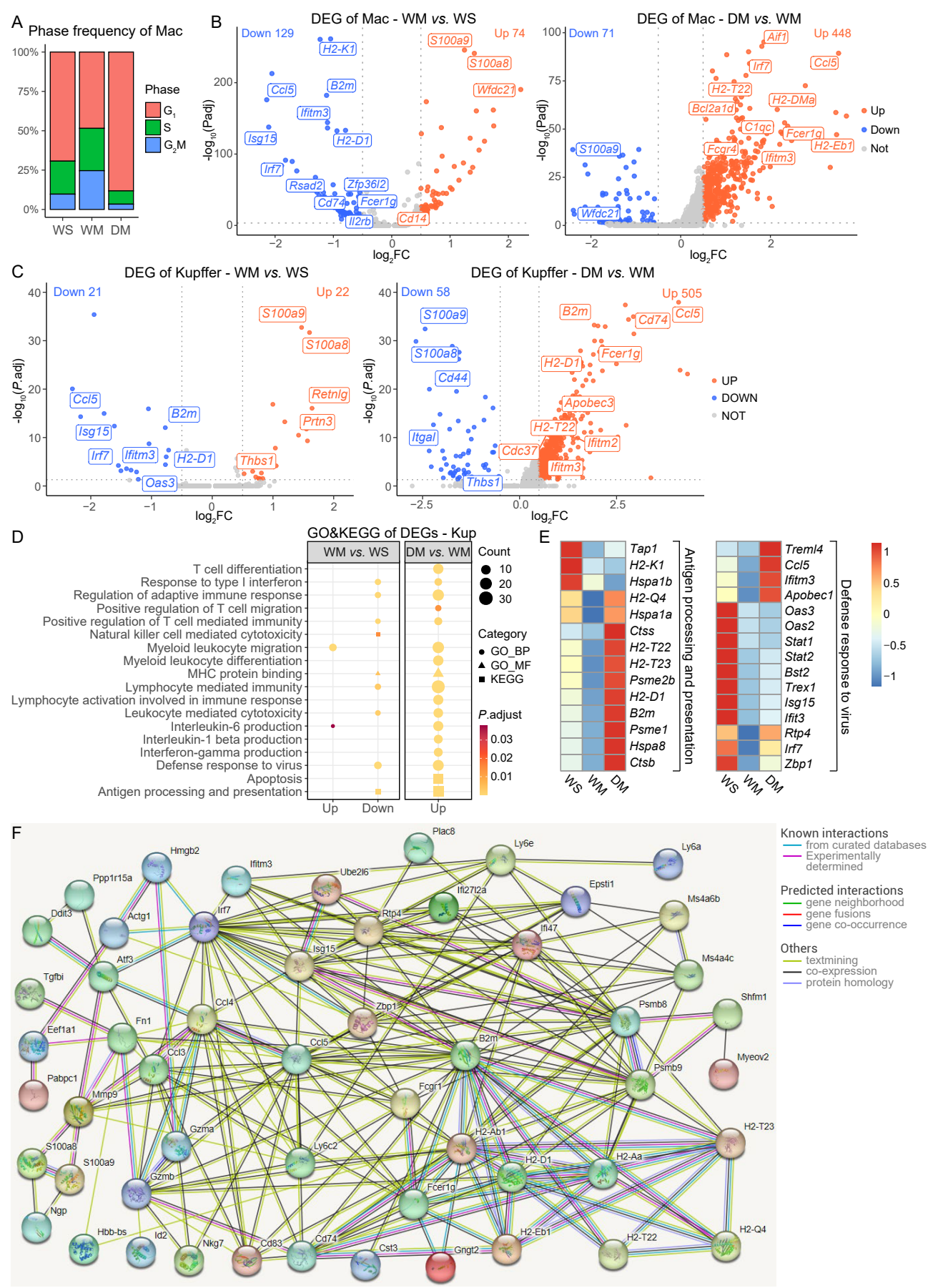

Supplement: qzae060_Supplementary_Data [file qzae060_supplementary_data.zip › Figure S4.pdf]

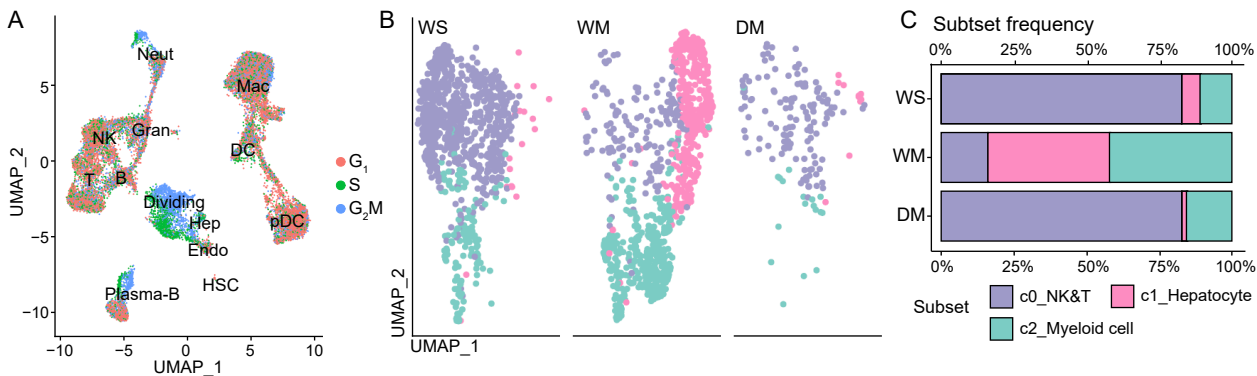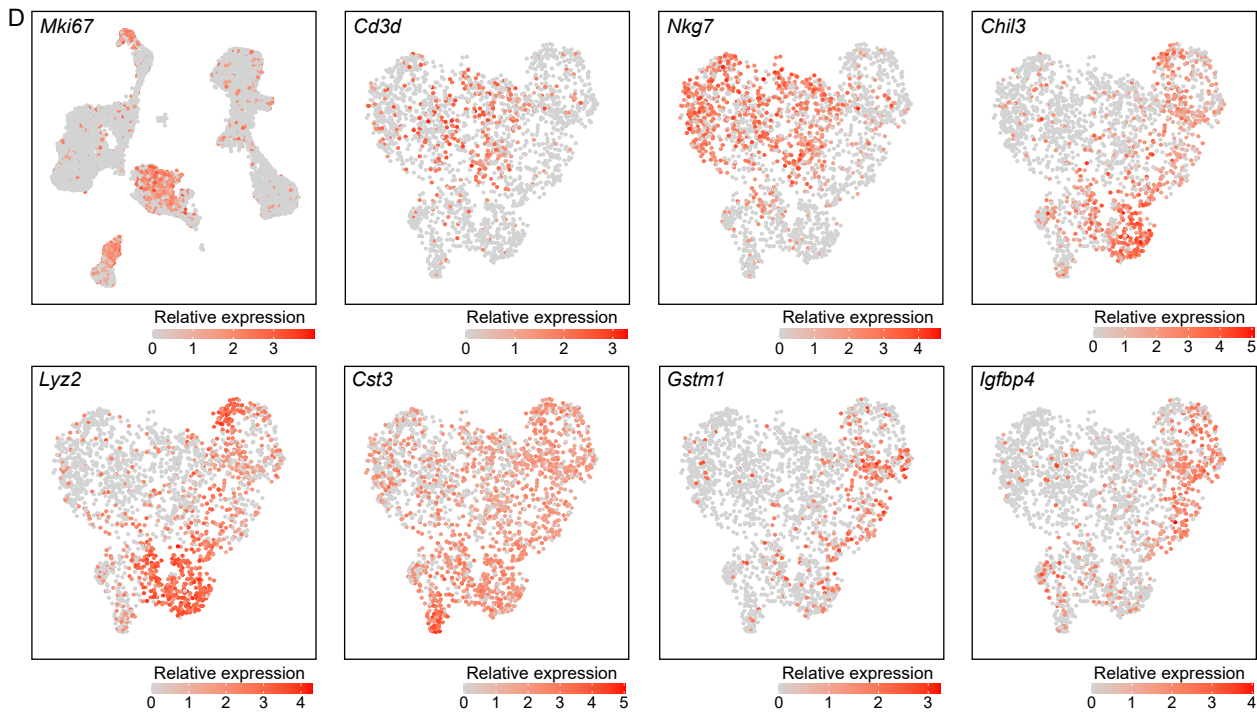

Supplement: qzae060_Supplementary_Data [file qzae060_supplementary_data.zip › Figure S2.pdf]

# GO&KEGG enrich of B subclusters markers

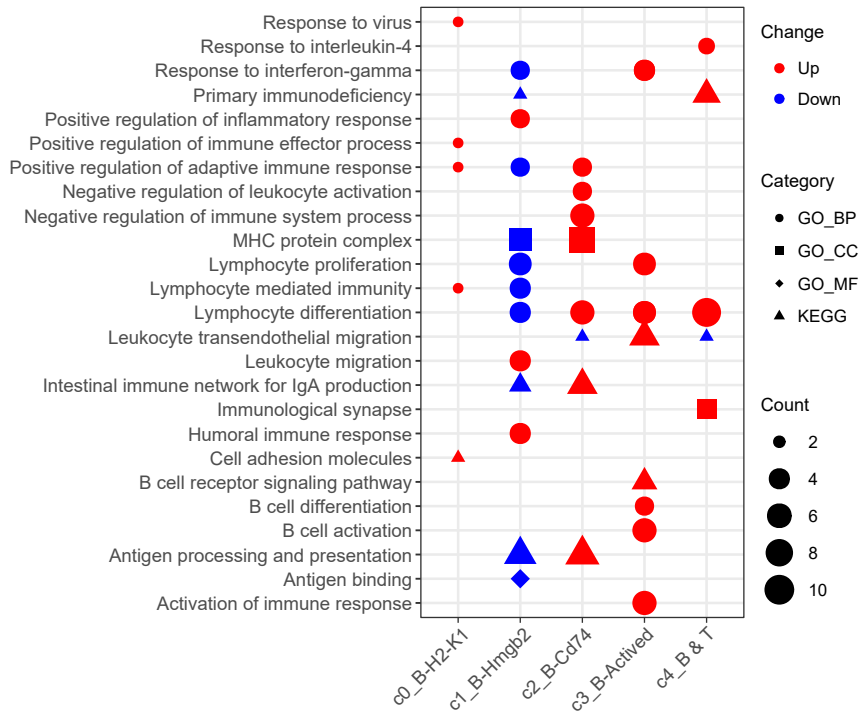

Supplement: qzae060_Supplementary_Data [file qzae060_supplementary_data.zip › Figure S6.pdf]
