# Supplementary material for: Single-cell RNA-seq Reveals the Inhibitory Effect of Methamphetamine on Liver Immunity with the Involvement of Dopamine Receptor D1
Source: Genomics Proteomics Bioinformatics. 2024 Aug 28;22(4):qzae060. doi: 10.1093/gpbjnl/qzae060 (PMC11576359; doi:10.1093/gpbjnl/qzae060)
Supplement: qzae060_Supplementary_Data [file Figure S5.pdf › Figure S5.pdf]

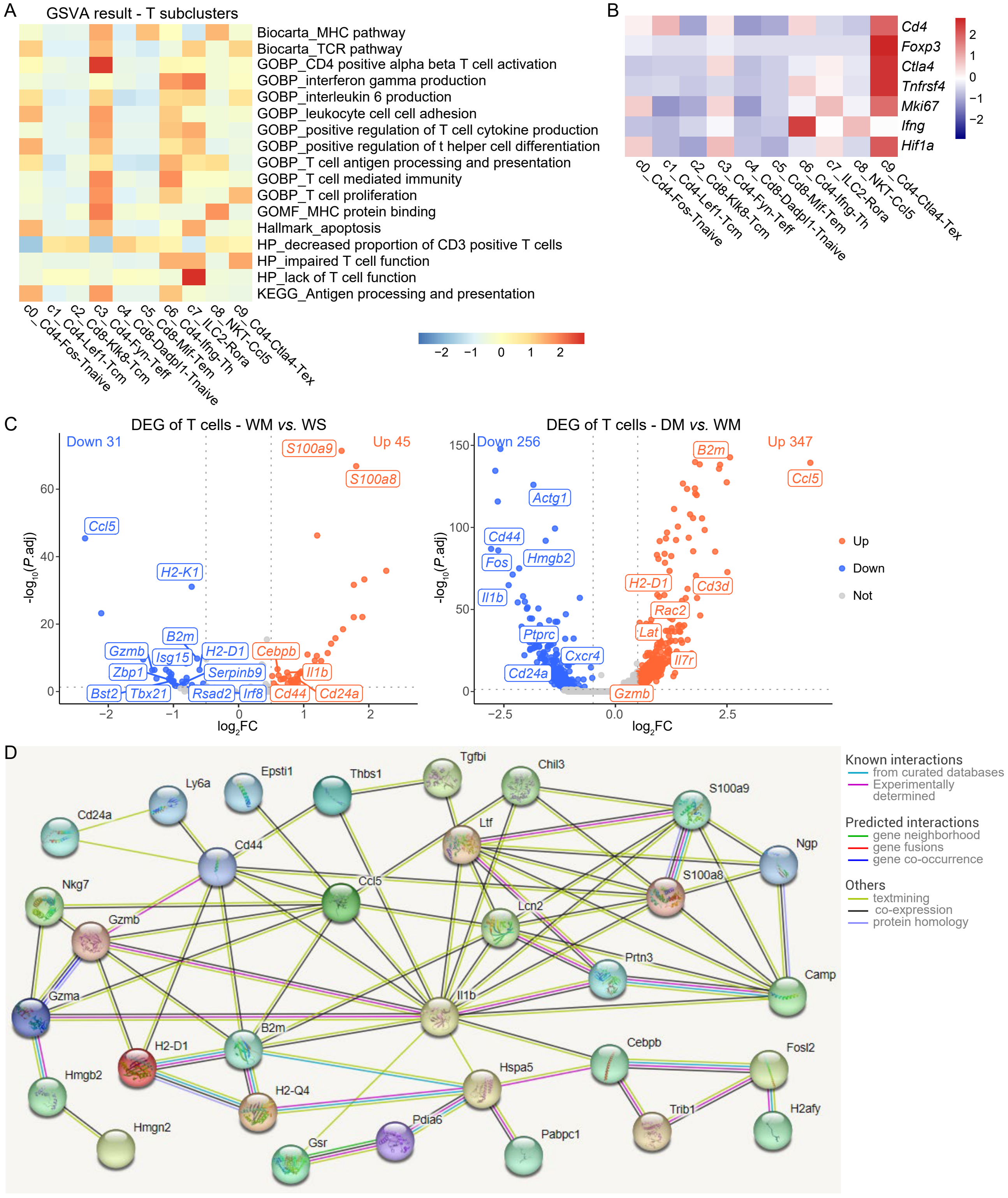
A
GSVA result - T subclusters
Biocarta_MHC pathway
Biocarta_TCR pathway
GOBP_CD4 positive alpha beta T cell activation
GOBP_interferon gamma production
GOBP_interleukin 6 production
GOBP_leukocyte cell cell adhesion
GOBP_positive regulation of T cell cytokine production
GOBP_positive regulation of t helper cell differentiation
GOBP_T cell antigen processing and presentation
GOBP_T cell mediated immunity
GOBP_T cell proliferation
GOMF_MHC protein binding
Hallmark_apoptosis
HP_decreased proportion of CD3 positive T cells
HP_impaired T cell function
HP_lack of T cell function
KEGG_Antigen processing and presentation
c0_Cd4-Fos-Tnaive
c1_Cd4-Lef1-Tcm
c2_Cd8-Klk8-Tcm
c3_Cd4-Fyn-Teff
c4_Cd8-Dadpl1-Tnaive
c5_Cd8-Mif-Tem
c6_Cd4-Ifng-Th
c7_ILC2-Rora
c8_NKT-Ccl5
c9_Cd4-Ctla4-Tex
−2 −1 0 1 2
B
Cd4
Foxp3
Ctla4
Tnfrsf4
Mki67
Ifng
Hif1a
2 1 0 −1 −2
C
DEG of T cells - WM vs. WS
Down 31
Up 45
S100a9
S100a8
Ccl5
H2-K1
B2m
Gzmb
Isg15
H2-D1
Cebpb
Zbp1
Serpinb9
Il1b
Bst2
Tbx21
Rsad2
Irf8
Cd44
Cd24a
-log10(P.adj)
log2FC
DEG of T cells - DM vs. WM
Down 256
Up 347
B2m
Ccl5
Actg1
Cd44
Fos
Hmgb2
Il1b
H2-D1
Cd3d
Rac2
Lat
Ptprc
Cxcr4
Il7r
Cd24a
Gzmb
Up
Down
Not
D
Tgfbi
Chil3
Epsti1
Thbs1
Ly6a
S100a9
Cd24a
Ltf
Cd44
Ngp
S100a8
Ccl5
Nkg7
Lcn2
Gzmb
Prtn3
Camp
Gzma
Il1b
B2m
H2-D1
Cebpb
Fosl2
Hspa5
Hmgb2
Trib1
H2afy
H2-Q4
Pdia6
Hmgn2
Pabpc1
Gsr
Known interactions
from curated databases
Experimentally determined
Predicted interactions
gene neighborhood
gene fusions
gene co-occurrence
Others
textmining
co-expression
protein homology
